# Distinct signaling routes mediate intercellular and intracellular rhizobial infection in *Lotus japonicus*

**DOI:** 10.1093/plphys/kiaa049

**Published:** 2020-12-04

**Authors:** Jesús Montiel, Dugald Reid, Thomas H Grønbæk, Caroline M Benfeldt, Euan K James, Thomas Ott, Franck A Ditengou, Marcin Nadzieja, Simon Kelly, Jens Stougaard

**Affiliations:** 1 Department of Molecular Biology and Genetics, Aarhus University, DK-8000, Aarhus C, Denmark; 2 The James Hutton Institute, Invergowrie, Dundee, DD2 5DA, UK; 3 Cell Biology, Faculty of Biology, University of Freiburg, 79104 Freiburg, Germany

## Abstract

Rhizobial infection of legume roots during the development of nitrogen-fixing root nodules can occur intracellularly, through plant-derived infection threads traversing cells, or intercellularly, via bacterial entry between epidermal plant cells. Although it is estimated that around 25% of all legume genera are intercellularly infected, the pathways and mechanisms supporting this process have remained virtually unexplored due to a lack of genetically amenable legumes that exhibit this form of infection. In this study, we report that the model legume *Lotus japonicus* is infected intercellularly by the IRBG74 strain, recently proposed to belong to the *Agrobacterium* clade of the *Rhizobiaceae*. We demonstrate that the resources available for *L. japonicus* enable insight into the genetic requirements and fine-tuning of the pathway governing intercellular infection in this species. Inoculation of *L. japonicus* mutants shows that *Ethylene-responsive factor required for nodulation 1* (*Ern1*) and *Leu-rich Repeat Receptor-Like Kinase* (*RinRK1*) are dispensable for intercellular infection in contrast to intracellular infection. Other symbiotic genes, including *nod factor receptor 5 (NFR5)*, *symbiosis receptor-like kinase (SymRK)*, Ca^2+/^*calmodulin dependent kinase (CCaMK), exopolysaccharide receptor 3 (Epr3), Cyclops*, *nodule inception (Nin)*, *nodulation signaling pathway 1 (Nsp1)*, *nodulation signaling pathway 2 (Nsp2)*, *cystathionine-β-synthase (Cbs)*, and *Vapyrin* are equally important for both entry modes. Comparative RNAseq analysis of roots inoculated with IRBG74 revealed a distinctive transcriptome response compared with intracellular colonization. In particular, several cytokinin-related genes were differentially regulated. Corroborating this observation, *cyp735A* and *ipt4* cytokinin biosynthesis mutants were significantly affected in their nodulation with IRBG74, whereas *lhk1* cytokinin receptor mutants formed no nodules. These results indicate a differential requirement for cytokinin signaling during intercellular rhizobial entry and highlight distinct modalities of inter- and intracellular infection mechanisms in *L. japonicus*.

## Teaser

Genetics and transcriptomics uncovers modalities of intercellular infection in legume-rhizobium symbiosis.

## Introduction

Legumes constitute a large and diverse plant family and most legumes are able to develop nitrogen-fixing root nodules in symbiosis with soil bacteria commonly referred to as rhizobia. Bacterial infection of roots and root nodules through intracellular infection threads (ITs) has been extensively researched in the model legumes *Lotus japonicus* (a relative of birdsfoot trefoil) and barrel medic (*Medicago truncatula*), as well as crop legumes like soybean (*Glycine max* L.), pea (*Pisum sativum* L.), and common bean (*Phaseolus vulgaris* L.). However, an alternative mechanism of intercellular infection is widespread in different genera of the Fabaceae family (Sprent et al., 2017) and proposed to represent an ancient and less sophisticated mechanism of rhizobial colonization (Sprent, 2007). In this process, rhizobia invade the legume roots between epidermal/root hair cells or by crack entry, during the protrusion of lateral roots (Coba de la Peña et al., 2017). Intercellular infection processes have been described in detail by microscopy in different legumes, such as *Mimosa*, *Neptunia*, *Stylosanthes*, *Cytisus*, and *Lupinus* (de Faria et al., 1988; James et al., 1992; Subba-Rao et al., 1995; Vega-Hernandez et al., 2001; Gonzalez-Sama et al., 2004; Goormachtig et al., 2004). Special attention has been dedicated to characterize the histology of intercellular infection and nodulation processes in *Arachis hypogaea*, *Aeschynomene* spp. and the semiaquatic legume *Sesbania rostrata* (Chandler, 1978; Boogerd and Van Rossum, 1997; Bonaldi et al., 2011; Ibañez et al., 2017). Under flooded conditions, rhizobial colonization takes places via infection pockets in *S. rostrata*, formed by a cell death process that depends on nodulation factors (NFs), perception, and localized formation of reactive oxygen species (D'Haeze et al., 2003). From such infection pockets, cortical ITs are formed and migrate to the nodule primordium, where the bacteria are released from the ITs and colonize the nodule cells in symbiosomes (Capoen et al., 2010). Unlike intracellular colonization, the *symbiosis receptor-like kinase* (*SymRK*) gene and the Ca^2+^/calmodulin-dependent protein kinase (*CCaMK*) gene are dispensable for intercellular infection in *S. rostrata* by *Azorhizobium caulinodans*. However, these genes are required for the subsequent intracellular cortical ITs (Capoen et al., 2005, 2009). In peanut (*Arachis hypogaea*), bradyrhizobia enter through the middle lamellae of two adjacent root hairs and spread intercellularly between epidermal and cortical cells. In parallel, adjacent axillary root hair basal cells become enlarged and infected by the microsymbiont (Chandler, 1978; Boogerd and Van Rossum, 1997; Guha et al., 2016). Both nodule formation and nodule cell colonization require proper exopolysaccharide production by rhizobia (Morgante et al., 2007). Similarly, an NF-independent nodulation program has been described in certain *Aeschynomene* spp. (Giraud et al., 2007). Currently, the *Aeschynomene evenia–Bradyrhizobium* symbiosis has been employed to study the molecular genetics of this unusual NF-independent symbiosis (Arrighi et al., 2012). Recent findings show that during this peculiar mechanism, several components of the NF-dependent process are also recruited, such as SYMRK, CCaMK, and the *Histidine Kinase* (HK1) cytokinin receptor (Fabre et al., 2015). Additionally, the structural requirements to perceive NF in *S. rostrata* are more permissive in intercellular infection compared with the intracellular infection (Goormachtig et al., 2004). Intercellular infection occurs in several *Sesbania* spp. by IRBG74 (Cummings et al., 2009), a nodulating strain that belongs to the *Agrobacterium* clade of the *Rhizobiaceae* (Aguilar et al., 2016), which is also able to colonize rice and Arabidopsis roots as an endophyte (Biswas et al., 2000; Mitra et al., 2016; Zhao et al., 2017). Therefore, a better understanding of intercellular colonization could facilitate the engineering of nonlegume crops for colonization by nitrogen-fixing bacteria.

In *Lotus*, NFs are recognized in the plasma membrane of the root hairs by nod factor receptors (NFR1, NFR5, and NFRe; Madsen et al., 2003; Radutoiu et al., 2003; Murakami et al., 2018). A compatible recognition leads to rhizobial attachment to the root hair tip, promoting its curling to trap the bacteria within an infection pocket. This gives rise to the formation of an IT, a tubular structure with an inward growth that originates from invagination of the plasma membrane of the root hair. The IT follows a polar growth toward inner root cell layers, reaching the nodule primordia, formed by the activation of cell division in the cortical cells. The nodule primordia give rise to mature nodules, wherein the bacteria are released from the IT into symbiosomes and differentiate to become nitrogen-fixing bacteroids (Downie, 2014).

The intracellular infection of rhizobia via ITs in the root hairs has been extensively investigated in *Lotus* and *M. truncatula* (Lace and Ott, 2018). In these legumes, the infection is orchestrated by several transcription factors, including nodule inception (*Nin*; Schauser et al., 1999; Marsh et al., 2007), *Nsp1*/*Nsp2* (Kalo et al., 2005; Heckmann et al., 2006), *Cyclops* (Yano et al., 2008; Singh et al., 2014), and *ERF* required for nodulation (*Ern1*; (Cerri et al., 2012; Kawaharada et al., 2017). The latter is required for activation of the expression of the cytokinin-biosynthesis genes *Ipt2* and *Log4* that are major contributors to the initial symbiotic cytokinin response in *Lotus* (Reid et al., 2017). Cytokinin is necessary for nodule organogenesis but plays a negative role during rhizobial invasion. In the cytokinin oxidase/dehydrogenase 3 mutant (*ckx3*), where cytokinin levels in the roots are increased, rhizobial infection is significantly reduced (Reid et al., 2016). In contrast, the roots of cytokinin receptor *Lhk1* mutants are hyperinfected by rhizobia (Murray et al., 2007). Recent reports show that additional signaling pathways play a positive role in infection, like the exopolysaccharide receptor EPR3 (Kawaharada et al., 2015) and the Leu-rich repeat receptor-like kinase (RINRK1; Li et al., 2019). In addition, several molecular components are required. Mutants disrupted in the E3 ligase *Cerberus* (Yano et al., 2009), the nodule pectate lyase *Npl1* (*Xie et al., 2012*) or *Arpc1*, *ScarN, Nap1, Pir1* involved in actin rearrangements (Yokota et al., 2009; Hossain et al., 2012; Qiu et al., 2015), show defects in IT development and abortion of the infection process. In *Medicago*, the IT localized cystathionine-β-synthase-like 1 (CBS1; Sinharoy et al., 2013), coiled-coil RPG protein (Arrighi et al., 2008) and Vapyrin (Murray et al., 2011) are crucial components of the root hair infectome (Liu et al., 2019).

Rhizobia cross the epidermal cell layer of legume roots intracellularly through root hair ITs, or by intercellular infection. The plant genetic basis for the latter is largely unexplored despite its estimated prevalence in 25% of all legumes and its engineering potential to achieve rhizobial colonization in nonlegume crops (Sprent, 2007; Oldroyd and Dixon, 2014). To understand and compare the genetic programs controlling intracellular and intercellular infection in *Lotus* roots, we have analyzed the infective capacity of *Rhizobium* sp. strain IRBG74, where a wide range of genetic, genomic, and transcriptomic resources are available. Crucial genes for both modalities of rhizobial infection were identified along with distinctive cellular, transcriptome, and genetic requirements for intercellular colonization.

## Results

### IRBG74 induces nitrogen-fixing nodules in *Lotus*

**Figure 1:**
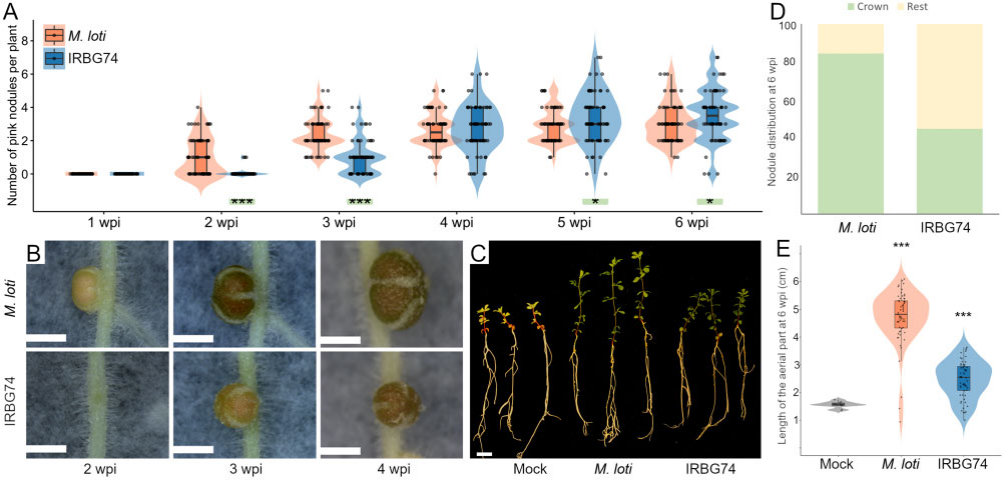
Nodulation phenotype of *Lotus* plants inoculated with *M. loti* or IRBG74. (**A**) Nodule numbers at 1–6 wpi. Mann–Whitney *U*-test of pink and total number of nodules (asterisks below the violin graphs indicates significant difference: *P < 0.05; ***P < 0.001) between Gifu plants inoculated with *M. loti* and IRBG74. (**B**) Images of nodules at 2–4 wpi with *M. loti* or IRBG74 (upper and lower panels, respectively). Scale bars = 1 mm. (**C**) Representative plants at 6 wpi with *M. loti*, IRBG74 or uninoculated (mock). Images were digitally extracted for comparison. Scale bar = 1 cm. (**D**) Nodule distribution in *Lotus* roots at 6 wpi with *M. loti* or IRBG74. (**E**) Shoot length of *Lotus* plants harvested at 6 wpi with *M. loti*, IRBG74 or uninoculated (mock). Student’s *t* test of length of the aerial part between mock-treated plants and inoculated with *M. loti* or IRBG74. *P < 0.05; **P < 0.01; ***P < 0.001; *n* ≥ 66. Violin boxplots: center line, median; box limits, upper and lower quartiles; whiskers, 1.5× interquartile range; points, individual data points.

The rhizobial strain IRBG74, infects *Sesbania cannabina* intercellularly (Cummings et al., 2009; Mitra et al., 2016) and interestingly it is also capable of colonizing *Oryza sativa* and *Arabidopsis thaliana* roots as an endophyte (Mitra et al., 2016; Zhao et al., 2017). In order to evaluate the infective capacity of IRBG74 in *Lotus*, nodulation kinetics was recorded from 1- to 6-week postinoculation (wpi) of the Gifu accession. As a control, *L. japonicus* seedlings were inoculated with its customary symbiont *Mesorhizobium loti* R7A that infects intracellularly. Nodule primordia were observed at 1 wpi on *Lotus* roots inoculated with *M. loti*, with mature pink nodules evident by 2 wpi (Figure 1A). IRBG74 also induced nodule organogenesis in *Lotus*, but the first nodule primordia were not observed until 2 wpi (Figure 1, A and B). The first mature pink nodules on plants inoculated with IRBG74 usually appeared at 3 wpi, but these were evidently smaller compared with the pink nodules induced by *M. loti* at the same time point (Figure 1B). In addition, the number of pink and total nodules was significantly lower at 2 and 3 wpi in plants inoculated with IRBG74 compared with plants inoculated with *M. loti* (Figure 1A;Supplemental Figure 1B), but after 4–6 wpi, the number of nodules on plants inoculated with *M. loti* and IRBG74 was comparable (Figure 1A;Supplemental Figure 1B). The delay in the IRBG74 nodulation was reflected by the distribution pattern of the nodules in the root system, since 45% of the nodules were found in the root crown (upper 2 cm of the root system), whereas plants inoculated with *M. loti* had 84% of the nodules in this segment of the root (Figure 1, C and D). Nodules induced by IRBG74 were pink and the plant shoots were green (Figure 1, B and C), indicative of nitrogen fixation. At 6 wpi, shoot lengths were significantly lower compared with plants inoculated with *M. loti*, reflecting the delayed nodule formation, but higher compared with mock-treated plants (Figure 1, C and E). These results show that IRBG74 is able to induce nitrogen-fixing nodules in *Lotus*, albeit with a delay.

To determine if the delay in the nodulation program by IRBG74 was restricted to the accession Gifu, the nodulation kinetics was recorded at 1–3 wpi with IRBG74 in three different *Lotus* ecotypes (MG134, MG144, and MG145; Hashiguchi et al., 2012), testing in parallel plants inoculated with *M. loti*. The first nodule primordia were observed at 1 wpi with *M. loti* in the different *Lotus* accessions, whereas in response to IRBG74 primordia emerged from the second week. This analysis shows that the delay in the nodulation process by IRBG74 is not restricted to accession Gifu (Supplemental Figure 1A).

### *Lotus* is intercellularly infected by IRBG74

**Figure 2:**
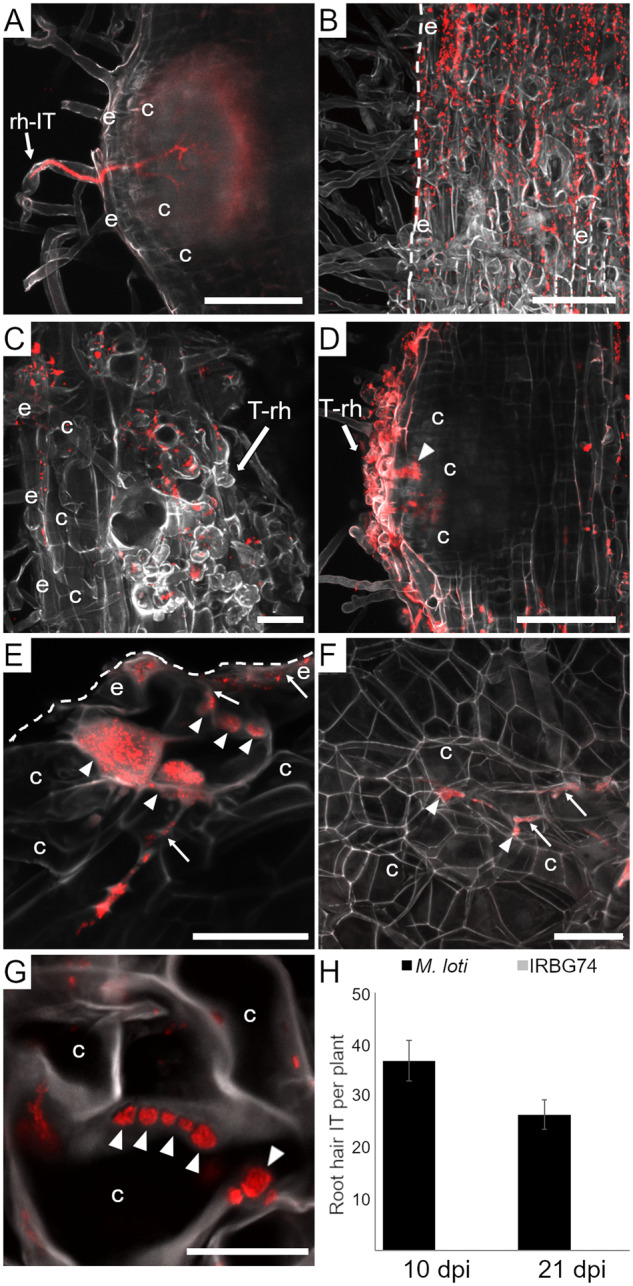
Intracellular and intercellular rhizobial infection of *L. japonicus* roots. (**A**) *L. japonicus* root with root hair ITs at 1 wpi with *M. loti*-DsRed. (**B**) IRBG74-DsRed at 1 wpi associated with the surface of the root epidermal cells (boundaries highlighted with dashed lines). (**C**–**G**) Nodule primordia of 2–3 wpi with IRBG74-DsRed at progressive colonization and developmental stages. Highly deformed and twisted root hairs in emerging nodule primordia (**C** and **D**). Intercellular infection of IRBG74-DsRed at the root surface (**E**) is followed by infection pocket formation in subepidermal cell layers and intercellular progression (**E**–**G**). Scale bar, 50 *µ*m (**A**–**D**) and 20 *µ*m (**E**–**G**). (**H**) Number of root hair ITs per plant at different time points (Error bars mean SE; *n* = 14). rh-IT, root hair IT; T-rh, twisted root hairs; e, epidermis; c, cortex; arrows, intercellular infection; arrowheads, infection pockets. Dashed lines indicate the surface and some of the boundaries of root epidermal cells in **B** and **E** (see Supplemental Movie 1).

The delay in organogenesis following inoculation with IRBG74 compared with *M. loti* prompted us to explore the infection process. For this purpose, the constitutive DsRED expressing plasmid pSKDSRED was transformed into IRBG74 in order to monitor the early infection process by confocal microscopy. *Mesorhizobium loti*-DsRed was used as a control. Infection and nodule organogenesis were unaltered with these engineered strains. Typical intracellular ITs in long root hairs were abundant at 7-d postinoculation (dpi) with *M. loti*-DsRed (Figure 2A). In contrast, no IRBG74-DsRed ITs were observed. IRBG74 was attached to the surface of the roots, mainly associated with the boundaries of the epidermal and root hair cells (Figure 2, B–D; Supplemental Video 1). A detailed and quantitative inspection revealed an average of 35 root hair ITs in response to *M. loti*-DsRed, whereas none were found in the entire root system of plants inoculated with IRBG74-DsRed at 10 and 21 dpi (Figure 2H). IRBG74-DsRed was observed on the epidermis of emergent nodule primordium, associated to highly deformed and twisted root hairs (Figure 2, C and D; Supplemental Movie S1). IRBG74, therefore, elicits a root hair response but no root hair ITs were observed in these deformed root hairs. In nodule primordia at a more advanced developmental stage, IRBG74-DsRed infected the epidermal cells intercellularly (Figure 2, E). The intercellular infection was followed by the formation of infection pockets accumulating IRBG74 in the subepidermal root cell layers (Figure 2, E–G). From these infection pockets, rhizobial colonization progressed either transcellularly or intercellularly into inner root cell layers (Figure 2, E and F; Supplemental Movie 1).

**Figure 3:**
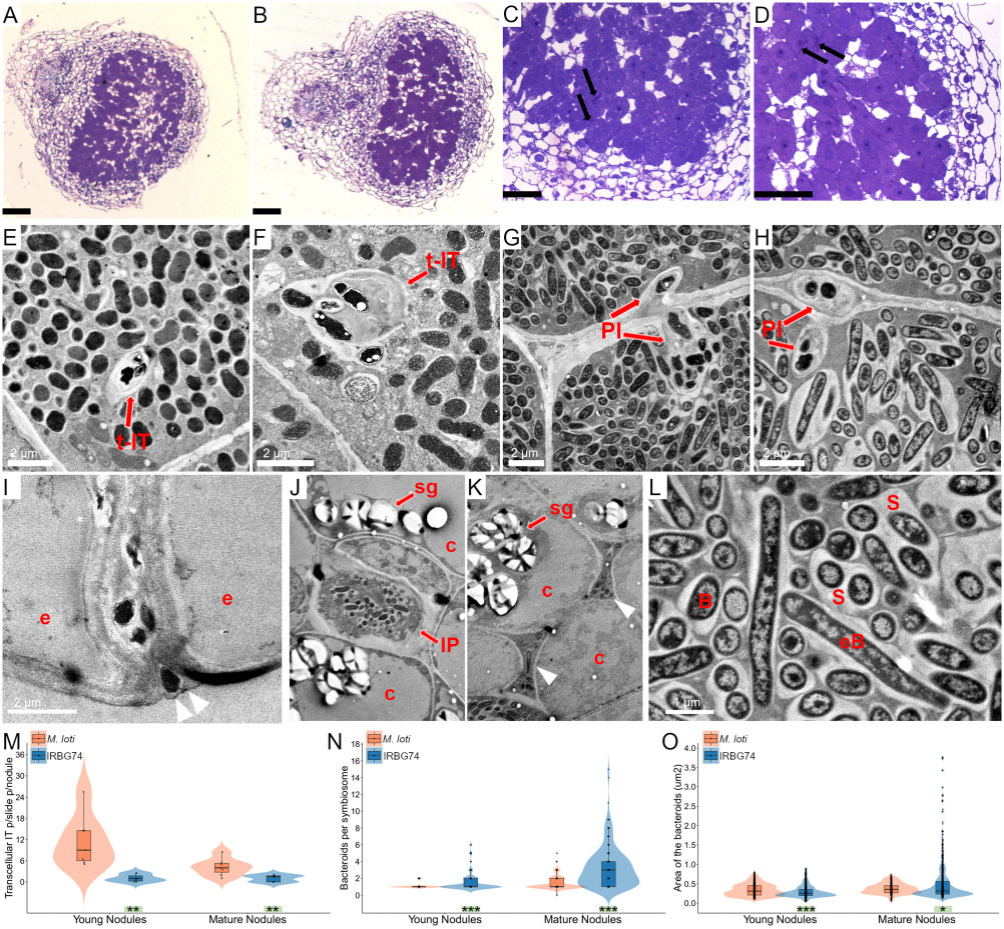
Histology and intercellular path of *L. japonicus* nodules colonized by IRBG74. Nodule sections stained with toluidine blue and visualized by light microscopy illustrate the structure and presence of transcellular ITs in young (**A** and **C**) and mature nodules (**B** and **D**) colonized by *M. loti* (A and B) and IRBG74 (C and D). TEM micrographs of young (**E** and **G**) and mature nodules (**F** and **H**) reveal transcellular ITs (E and F) and peg-infection (G and H) by *M. loti* and IRBG74, respectively. IRBG74 infection between the epidermal cells of a young nodule (**I**) is followed by infection pocket formation (J) and intercellular progression in the cortex (K). (**L**) Magnification of a nodule cell with symbiosomes and elongated IRBG74 bacteroids. Number of transcellular ITs per nodule (**M**), number of bacteroids per symbiosome (**N**) and area of the bacteroids in young and mature nodules (**O**) colonized by *M. loti* and IRBG74. Student’s *t* test (M) and Mann–Whitney *U*-test (N and O). *P<0.05; ** *P*<0.01; and ***P<0.001. Violin boxplots: center line, median; box limits, upper and lower quartiles; whiskers, 1.5× interquartile range; points, individual data points. Black arrows, transcellular ITs (t-IT); Red arrows, highlight different structures. Arrowheads, intercellular colonization; IP, infection peg; PI, peg-infection; e, epidermis; c, cortex; sg, starch granules; B, bacteroids; eB, elongated bacterioids. Scale bar, 100 *µ*m (A and B) and 50 *µ*m (C and D).

The confocal microscopy analysis revealed an intercellular infection of IRBG74 in *Lotus* roots. To characterize the progression of the IRBG74 infection process in more detail, the histology of young and mature nodules was analyzed by light microscopy and compared with nodules of similar developmental stage induced by *M. loti* at 3 wpi. Symbiosome-containing nodule cells and transcellular ITs were observed in both young and mature nodules of *Lotus* with both rhizobial strains (Figure3, A–D; Supplemental Figure 2, A–C). These structures were remarkably more numerous in nodules colonized by *M. loti*, compared with IRBG74, particularly in young nodules (Figure 3M). To discern differences in infection mechanisms and structures with more resolution, we decided to evaluate the bacteroid occupancy and infection within the nodule cells by transmission electron microscopy (TEM). Nodule cells were successfully occupied by *M. loti* and IRBG74 in young and mature nodules (Figure 3, E–H). The symbiosomes commonly contained one or two bacteroids in young nodules colonized by *M. loti* and IRBG74 (Figure 3, E–G and N). However, mature nodules infected by IRBG74 contained large symbiosomes with multiple bacteroids (Figure 3, H, L, N, and O). Similar to what was observed by confocal and light microscopy, transcellular ITs were found in nodules inoculated with this symbiont (Supplemental Figure 2E). Additionally, the TEM micrographs clearly showed a contrasting infection mechanism between nodules colonized by *M. loti* and IRBG74. Only the typical transcellular ITs were abundant in nodules infected by *M. loti* (Figure 3, E and F). In contrast, butterfly-shaped infection pegs, originated from the intercellular space of nodule cells was observed with IRBG74 (Figure 3, G and H). TEM images also documented progression of IRBG74 infection, from the intercellular infection site in the epidermis of young nodules (Figure 3I;Supplemental Figure 2D), followed by the formation of infection pockets, where IRBG74 was accumulated both in these structures and the intercellular space between uninfected cells in the cortex (Figure 3, J and K; Supplemental Figure 2D), and ultimately colonization of infected cells via infection pegs or transcellular ITs (Figure 3G;Supplemental Figure 2, E and F). In summary, using complementary approaches, we found that IRBG74 transgresses the epidermis intercellularly and forms infection pockets; subsequent progression is either via transcellular ITs or intercellularly, resulting in the formation of symbiosomes by peg infection. Thus, representing a substantial difference in infection mechanism compared with the “standard” intracellular *M. loti* infection process in *Lotus*.

### *rinrk1* and *ern1* mutants show contrasting symbiotic phenotypes with IRBG74 and *M. loti*

**Figure 4:**
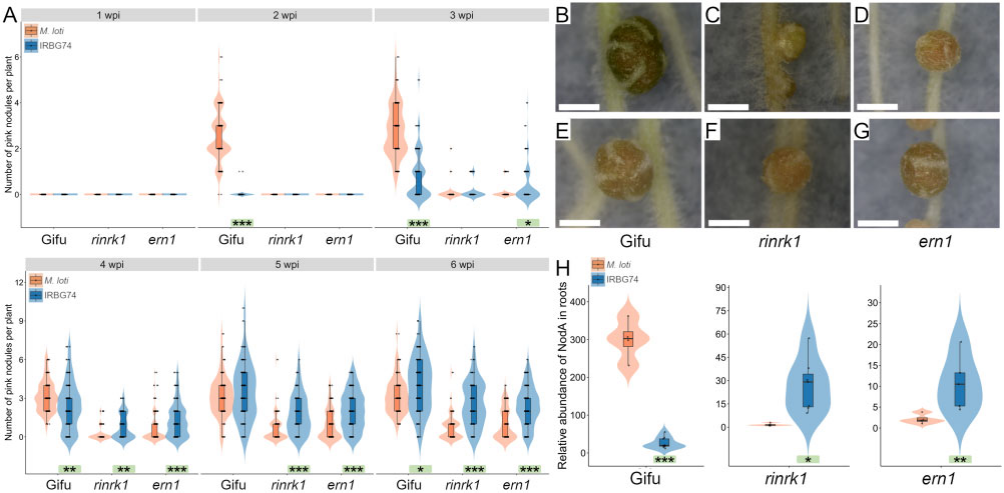
Nodulation phenotype of *rinrk1* and *ern1* mutants. **(A**) Nodulation kinetics from 1 to 6 wpi with *M. loti* or IRBG74 in *rinrk1* and *ern1*. Mann–Whitney *U*-test of pink nodules between plants inoculated with *M. loti* or IRBG74 in the same genetic background. *n* = Gifu ≥ 59; *rinrk1 *≥* *49; *ern1 *≥* *77. Representative images of nodules at 4 wpi with IRBG74 (**E**–**G**) or *M. loti* (**B**, **C**, and **D**) from Gifu (B and E), *rinrk1* (C and F) and *ern1* (D and G). Scale bar 1 mm. H, rhizobial occupancy of *L.* japonicus roots by qPCR. The abundance of *M. loti* and IRBG74 *nodA* gene was determined by qPCR using total DNA isolated from Gifu, *rinrk1* and *ern1* roots at 4 wpi with *M. loti* or IRBG74. The *nodA* accumulation was normalized to the LotjaGi1g1v0152000 gene abundance. Student’s *t* test of nodA abundance between roots inoculated with *M. loti* and IRBG74 in the same genetic background. * *P*<0.05; **P<0.01; and *** *P*<0.001. Violin boxplots: center line, median; box limits, upper and lower quartiles; whiskers, 1.5× interquartile range; points, individual data points.

Root infection and nodule organogenesis are highly coordinated multistep processes and nodule organogenesis is affected in mutants interrupted in rhizobial infection (Oldroyd and Downie, 2008; Madsen et al., 2010). Nodulation kinetics was, therefore, a suitable readout to determine the genetic dependency of the *Lotus*-IRBG74 intercellular process. IRBG74- and *M. loti*-induced nodulations of a set of previously identified *Lotus* symbiotic mutants were scored at 1–6 wpi. First, mutants affected in the symbiotic receptor genes *Nfr5*, *SymRK*, *RinRk1*, and *Epr3* were tested. Both *M. loti* and IRBG74 were unable to form nodules in the *nfr5* and *symrk* plants (Supplemental Table 2), indicating that IRBG74 nodulation is NF-dependent and requires functional NF receptors for recognition of the NF produced by IRBG74 (Crook et al., 2013; Poinsot et al., 2016) to trigger downstream signal transduction. This latter was further confirmed by the Nod^−^ phenotype observed in *Lotus* plants inoculated with an IRBG74 *nodA* mutant strain (Supplemental Table 2). However, the nodulation performance of the *rinrk1* mutant was different between plants inoculated with IRBG74 and *M. loti*. In *rinrk1* plants inoculated with *M. loti*, many white uninfected nodules were formed, with very few pink nodules at the different time points analyzed (Figure 4, A and C; Supplemental Figure 1C). In contrast, this hypernodulated-uninfected phenotype was not observed with IRBG74. The *rinrk1* plants infected by IRBG74 developed similar numbers of nodule structures to w.t. plants inoculated with *M. loti* or IRBG74 at 4–6 wpi, the majority of them pink, indicating effective rhizobial colonization and symbiosis (Figure 4, A and F; Supplemental Figure 1C). The number of pink nodules was significantly higher in plants infected by IRBG74 at 3–6 wpi compared with plants colonized by *M. loti*, indicating that intercellular infection was not impaired in the *rinrk1* mutant. The role of the expolysaccharide receptor *EPR3* is apparently important for both types of infection in *L. japonicus*, since a comparable delayed nodulation phenotype of the *epr3* mutant was observed following inoculation with *M. loti* or IRBG74 (Supplemental Figure 3A).

Since the infection process is also controlled by several transcriptional regulators, the role of *Nin*, *Cyclops*, *Ern1*, *Nsp1*, and *Nsp2* were tested with IRBG74. The transcription factors *Nin*, *Nsp1*, and *Nsp2* are indispensable for the symbiotic process established by IRBG74 and *M. loti*, since the mutants affected in these genes were unable to form nodules (Supplemental Table 2). The nodulation phenotype of *cyclops* plants inoculated with IRBG74 and *M. loti* was similar, and only uninfected white nodules developed (Supplemental Figure 3, B). However, a clear difference was found in the symbiotic performance of the *ern1* mutant. The first pink nodules appeared at 4 wpi with *M. loti* (Figure 4, A and D), whereas in the presence of IRBG74, these were detected at 3 wpi with fully developed pink nodules at 4 wpi (Figure 4, A and G). This is the reverse of the kinetics shown by w.t. plants wherein pink nodules emerged at 2 and 3 wpi with *M. loti* and IRBG74, respectively (Figure 1, A and B). Additionally, both the total number of nodules and pink nodules were higher in *ern1* mutants with IRBG74 at 3–6 wpi, compared with *M. loti* (Figure 4A;Supplemental Figure 1D). This difference in the symbiotic performance suggests a distinct genetic program promoting the intercellular colonization by IRBG74 in *Lotus*.

Both *ern1* and *rinrk1* mutants showed a greater ability to develop pink nodules in response to IRBG74 inoculation compared with *M. loti*. To assess if this response was accompanied by rhizobial infection, the root endosphere colonization was measured. The abundance of the rhizobial *nodA* gene was determined by quantitative real-time PCR, using total DNA extracted from roots at 4 wpi with *M. loti* and IRBG74 (Figure 4H). This analysis revealed that Gifu roots colonized by *M. loti* contained greater *nodA* abundance than plants inoculated with IRBG74: 299 versus 26 relative abundance units (rau), respectively. However, both the *rinrk1* and *ern1* mutants showed significantly lower *nodA* abundance in roots colonized by *M. loti* compared with roots inoculated with IRBG74. Interestingly, Gifu and *rinrk1* roots infected by IRBG74 showed comparable levels of *nodA* abundance in the roots, with 26 and 27 rau, respectively (Figure 4H). These results confirm a better colonization of *rinrk1* and *ern1* mutants through intercellular infection by IRBG74 compared with the root hair infection by *M. loti*.

### Nodulation by IRBG74 is negatively impacted in root hair IT mutants

In *Lotus*, several genes participating in a wide variety of molecular processes have been described as important for IT progression. For instance, the *Lotus* mutants affected in the U-box protein *Cerberus* (Yano et al., 2009), the nodule pectate lyase *Npl1* (Xie et al., 2012) or the cytoskeleton component *ScarN* (Qiu et al., 2015), show defects in IT growth and progression. The contribution of these genes to the intercellular infection of IRBG74 was therefore assessed. These mutants were characterized by the formation of a high proportion of white nodules both with *M. loti* and IRBG74 (Supplemental Figure 3, C–E), and few pink nodules were developed in the *npl1* and *scarN* mutants (Supplemental Figure 2, D and E). In all these mutants, the number of nodule structures was reduced when IRBG74 was used as inoculum (Supplemental Figure 3, C–E).

In *Medicago*, IT development has been shown to require the function of the *Vapyrin*, *RPG*, and *Cbs* genes. Since the participation of these genes has not been reported in *Lotus*–rhizobia symbiosis, their homolog counterparts were identified in the *Lotus* genome (https://lotus.au.dk/). For *Vapyrin*, two homologous genes were named *LjVpy1* (LotjaGi2g1v0091200) and *LjVpy2* (LotjaGi1g1v0646300; Supplemental Table 1), encoding proteins with 77% and 69% identity to the *Mt*VPY protein, respectively. Similarly, two genes encoding proteins with 61% and 31% amino acid identities to *Mt*RPG were named *Lj*RPG (LotjaGi5g1v0253300) and *Lj*RPG-like (LotjaGi5g1v0086600), respectively (Supplemental Table 1). The *Lj*CBS found (LotjaGi2g1v0126500) showed 78% of protein similarity to *Mt*CBS. Using the LORE1 database, mutant lines with LORE1 insertions were identified and genotyped to obtain homozygous mutant plants. A delay in nodule organogenesis was observed for all the mutants inoculated with *M. loti*, reflected by a reduced number of nodules within the first week after rhizobial inoculation (Supplemental Figure 3, F–J). In response to IRBG74 inoculation, nodulation was also delayed in *vpy1* and *vpy2* (Supplemental Fig. 3, G and H), but not in the *rpg* and *rpg*-*l* mutants, where the number of nodules were similar to the w. t. plants at different time points (Supplemental Fig. 3, I and J). Based on the nodule numbers, *vpy1* plants were more severely impacted with IRBG74 (Supplemental Fig. 3G), whereas the *vpy2* mutant was more affected with *M. loti* (Supplemental Fig. 3H). However, at 5 and 6 wpi, both the total number of nodules and pink nodules, tended to be similar between the w.t. and mutants. Since *MtVpy* and *MtRPG* have been described as important for IT development in *Medicago*, the number of root hair ITs was recorded in mutants affected in these genes in *Lotus* at 1 wpi with *M. loti*-DsRed. A significant reduction of IT numbers of around 50% in the *vpy1*, *vpy2*, and *rpg* mutants compared with w.t. was observed (Supplemental Table 3).

### Dispensable role of ROS and ethylene in the *Lotus*-IRBG74 symbiosis

It has been described that ethylene plays a positive role in the intercellular infection and nodulation program in the *S. rostrata*–*Azorhizobium* symbiosis (D'Haeze et al., 2003), which occurs under flooded conditions while infection in aerated soil occurs through root hair ITs (Ndoye et al., 1994; Goormachtig et al., 1998). To determine the role of this phytohormone in the *Lotus*-IRBG74 symbiotic process, the nodulation kinetics of the double mutant insensitive to ethylene *ein2a ein2b* (Reid et al., 2018) was recorded. The first nodule structures appeared at 2 wpi in the *ein2a ein2b* mutant inoculated with IRBG74, a similar kinetics to that observed in w.t. plants. The mutant showed a hypernodulation phenotype in the subsequent weeks, with 6, 12, and 16 nodules in average per plant at 4–6 wpi, which contrasts with the average of 2–4 nodules per plant developed in the w.t. background. However, the total number of nodules induced by IRBG74 was considerably lower than *M. loti* (Supplemental Fig. 3K). In addition, ethylene production was lower in w.t. *Lotus* roots inoculated with IRBG74 in comparison to plants treated with *M. loti* (Supplemental Fig. 4). Taken together, these results suggest that ethylene is not playing a positive role in the infection and organogenesis program triggered by IRBG74 in *Lotus*, and more likely, acts as a negative regulator of these processes in a manner similar to intracellular infection.

In several legumes, it has been shown that reactive oxygen species (ROS) produced by respiratory burst oxidase homolog (RBOH) enzymes are required for intracellular and intercellular infection (Peleg-Grossman et al., 2007; Montiel et al., 2012; Montiel et al., 2016; Arthikala et al., 2017). To address the involvement of these compounds in the symbiotic process induced by IRBG74 in *Lotus*, homozygous mutant lines affected in two *Rboh* isoforms, *LjRbohE* (LotjaGi5g1v0224200) and *LjRbohG* (LotjaGi1g1v0771200), were obtained from the LORE1 database. *LjRbohE* and *LjRbohG* are putative orthologs of *MtRbohA* and *PvRbohB*, previously characterized genes, required for nodule functioning and rhizobial infection in *Medicago* and common bean, respectively (Marino et al., 2011; Montiel et al., 2012). In response to *M. loti* inoculation, both *rbohE* and *rbohG* showed a reduced number of nodule primordia and pink nodules at 1 wpi (Supplemental Fig. 3, L and M). However, in the ensuing weeks, these nodule structures attained similar numbers to w. t. plants at all timepoints tested. The nodulation kinetics of these *rboh* mutants in response to IRBG74 infection was comparable to the w.t. plants (Supplemental Fig. 3, L and M), which indicates that these genes are not playing an important role in the intercellular colonization by IRBG74 in *Lotus*.

### Intercellular infection by IRBG74 promotes a distinct transcriptional reprogramming

**Figure 5:**
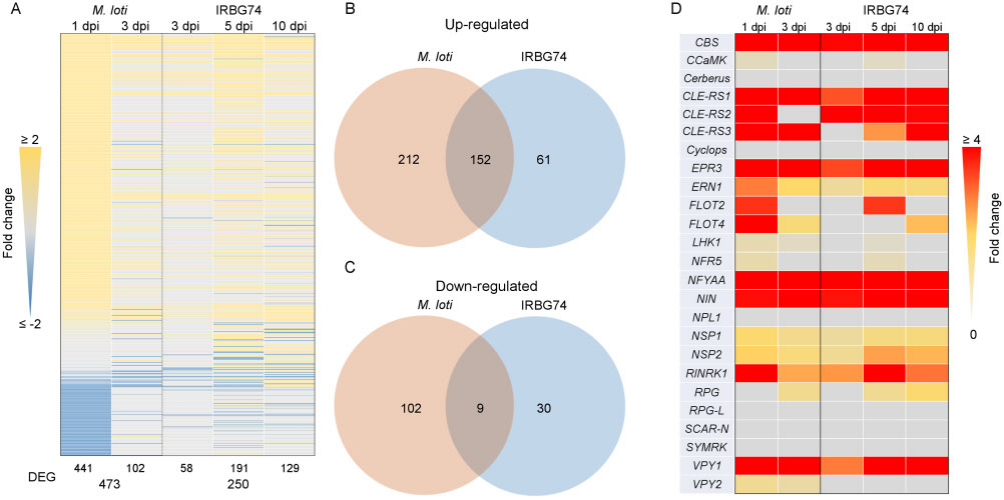
Comparison of the transcriptomic profile of *L. japonicus* roots during intracellular and intercellular rhizobial infection. (**A**) Heat-map expression of DEG [Log2 fold change {FC} ≥2 and a *P*-adjust <0.5] in *Lotus* roots after *M. loti* or IRBG74 inoculation. (**B** and **C**) Venn diagrams with total number (*M. loti*: 1 and 3 dpi; IRBG74: 3, 5, and 10 dpi) of the up/downregulated DEG during intracellular (*M. loti*) and intercellular (IRBG74) colonization. (**D**) Heat-map expression of known symbiotic genes significantly (*P*-adjust <0.5) induced after rhizobial perception.

The nodulation assays of the *Lotus* mutants inoculated with IRBG74 showed that the genetic dependencies for intercellular and intracellular infection modes differ. To further evaluate the signaling pathways involved in intercellular infection, RNAseq transcriptome data were collected and analyzed from the susceptible infection zone of IRBG74-inoculated *Lotus* roots at 3, 5, and 10 dpi, to cover the infection phase preceding nodule organogenesis. We decided to avoid the massive transcriptome program triggered during nodule development after 10 dpi, since this would hinder the identification of genes regulated during intercellular infection. These data were compared with available RNAseq information on the *Lotus* root susceptible zone and whole roots at 1 and 3 dpi with *M. loti*, respectively (Mun et al., 2016; Kamal et al., 2020), since these time points encompass different rhizobial infection stages in the root hairs, prior to the nodule organogenesis program. Due to the lack of information on the transcriptomic response in *Lotus* roots during intercellular infection and its notorious nodulation delay, first a low stringency analysis of differentially expressed genes (DEGs) was performed (*P*-adjust <0.5). In response to *M. loti* and IRBG74 inoculation, a total of 12,637 and 10,947 DEG were identified with these criteria, respectively (Supplemental Figure 5A; Supplemental Table 4). The largest transcriptome responses were observed at 1 dpi with *M. loti* (12,534) and at 10 dpi with IRBG74 (9,438) DEG (Supplemental Figure 5B). A more stringent analysis, with the DEG showing a LOG2FC ≥2, revealed that the most important transcriptome response was triggered at 5 dpi IRBG74 (Figure 5A). Interestingly, only 33% of the DEG by *M. loti* were similarly affected by IRBG74 and a large proportion of the up/downregulated genes during *M. loti* infection (314: sum of the up- and downregulated) were not similarly affected by IRBG74 (Figure 5, B and C). Additionally, the majority of the genes downregulated in response to IRBG74 inoculation were not repressed in the *M. loti* transcriptome (Figure 5, B and C). Through different stringency criteria, we found that IRBG74 induces a progressive increase in the number of DEG from 3 to 10 dpi, where only 1.3% (129 genes) showed >two-fold change at 10 dpi. In contrast, at 3 dpi, 17% (58) of the DEG had a more than two-fold change, showing a similar response to roots inoculated with *M. loti* at the same time point (Supplemental Figure 5C).

The nodulation kinetics of *Lotus* inoculated with IRBG74 revealed that nodule organogenesis was delayed with respect to plants inoculated with *M. loti*. To determine if this delay was linked to a deficient induction of the early symbiotic signaling pathway, the expression profile of several genes known to be involved in infection and/or nodule organogenesis were analyzed using the transcriptome data set. Most of the known symbiotic genes tested were induced by *M. loti* or IRBG74 (Figure 5D), but when the same time point was compared (3 dpi), several genes were less upregulated in response to IRBG74 inoculation (Figure 5D).

### Cytokinin signaling is differentially regulated in response to IRBG74

**Figure 6:**
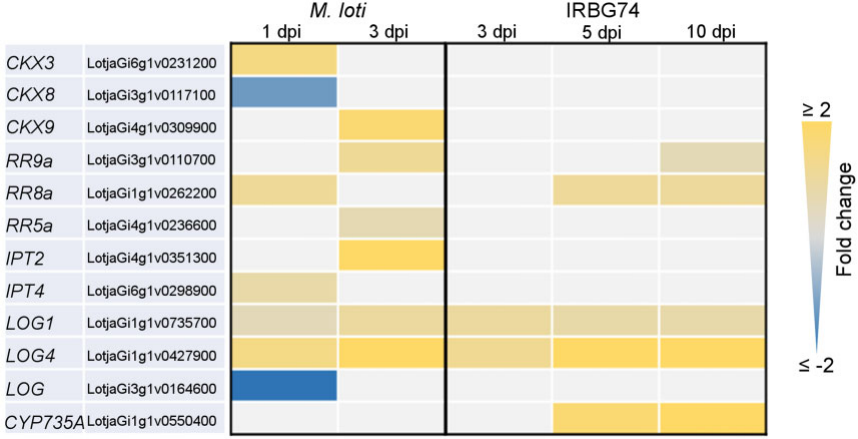
Heatmap of DEGs encoding cytokinin-related proteins in *L. japonicus* roots after rhizobial inoculation. The heatmap highlights the differences in the expression profile of DEG [log2-fold change {FC} ≥0.5, *P*-adjust <0.5) involved in the metabolism, perception, signaling and synthesis of cytokinins in response to *M. loti* or IRBG74 infection.

This work showed that *Ern1*, a transcription factor implicated in cytokinin signaling (Cerri et al., 2017; Kawaharada et al., 2017), has a less relevant role during intercellular infection by IRBG74. These results suggest that this mode of infection triggers a different transcriptional response of cytokinin-related genes compared with intracellular infection. To validate this hypothesis, a transcriptome heat-map was created for genes involved in cytokinin synthesis and regulation and significantly affected by *M. loti* or IRBG74 inoculation (Figure 6). This approach confirmed the different gene-expression response of several components of cytokinin regulation during IRBG74 infection. Particularly, genes encoding the cytokinin degrading enzymes *Ckx3*, *Ckx9*, the response regulator involved in cytokinin signaling *RR11a*, and the cytokinin biosynthesis gene *Ipt2* were poorly or insignificantly induced during intercellular infection, relative to their evident upregulation after *M. loti* inoculation (Figure 6). *Mesorhizobium loti* triggered a significant reduction in the expression levels of *Ckx2*, *Ckx8*, *Lhk2*, *RR3a*, *RR19*, and *Log*. However, most of these genes were not significantly downregulated by IRBG74 colonization. In contrast, the cytokinin biosynthesis gene *Cyp735a*, which converts iP to tZ type cytokinins, showed a strong upregulation after IRBG74 colonization at 5 and 10 dpi, whereas its gene expression was only slightly altered during intracellular infection at early timepoints (Figure 6).

### *Cyp735a, Ipt4*, and *Lhk1* are relevant players in the *Lotus*-IRBG74 symbiosis

**Figure 7:**
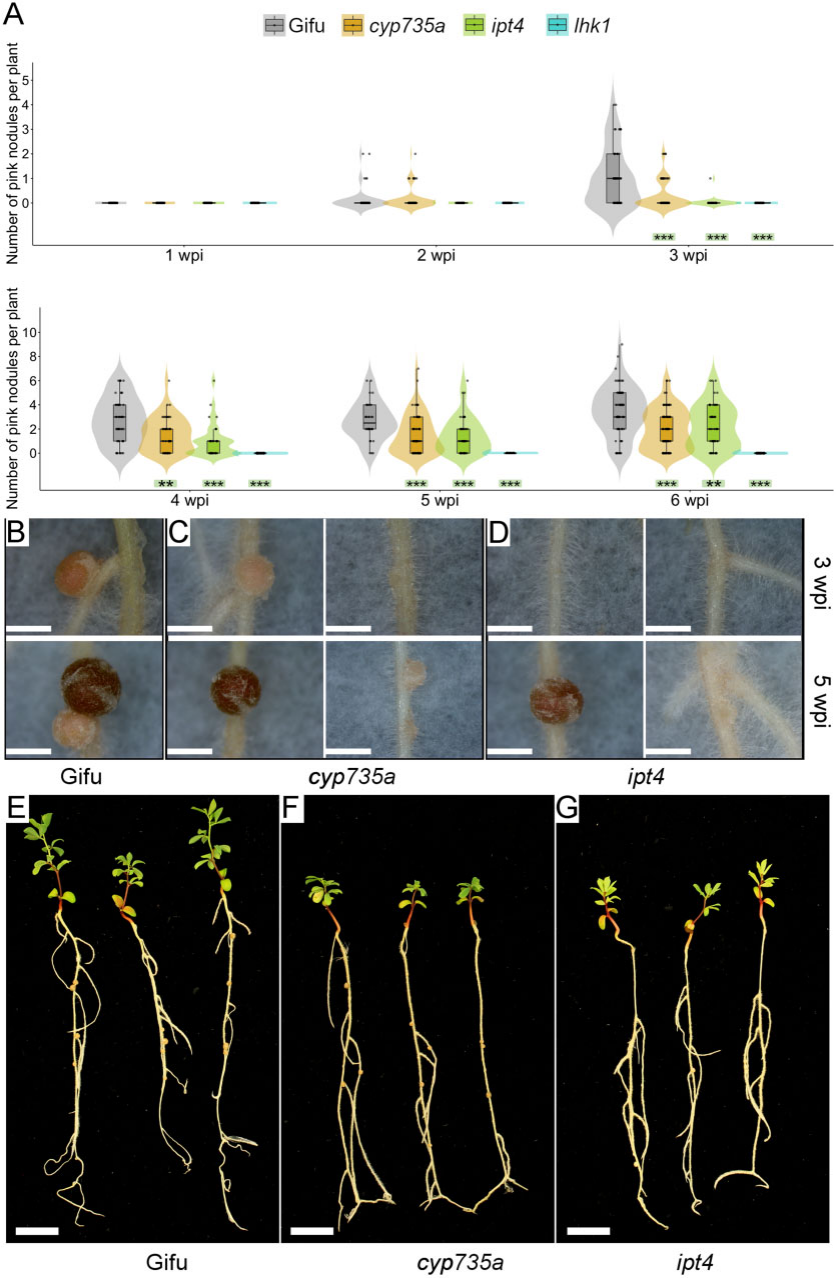
Symbiotic phenotype of *L. japonicus* mutants affected in cytokinin-related genes. (**A**) Nodulation kinetics of *cyp735a*, *ipt4*, and *lhk1* plants from 1 to 6 wpi with IRBG74. Mann–Whitney *U*-test of pink nodules. **P<0.01; ***P<0.001. *n* = 49 (Gifu), 86 (*cyp735a*), 53 (*ipt4*), and 55 (*lhk1*). Phenotype of nodules developed in wild-type Gifu (**B**), *cyp735a* (**C**) and *ipt4* (**D**) plants at 3 (upper panel) and 5 wpi (lower panel) with IRBG74. Representative images of wild type Gifu (**E**), *cyp735a* (**F**), and *ipt4* (**G**) plants at 6 wpi with IRBG74. Scale bar, 1 mm (B–D) and 1 cm (E–G). Violin boxplots: center line, median; box limits, upper and lower quartiles; whiskers, 1.5× interquartile range; points, individual data points.

**Figure 8:**
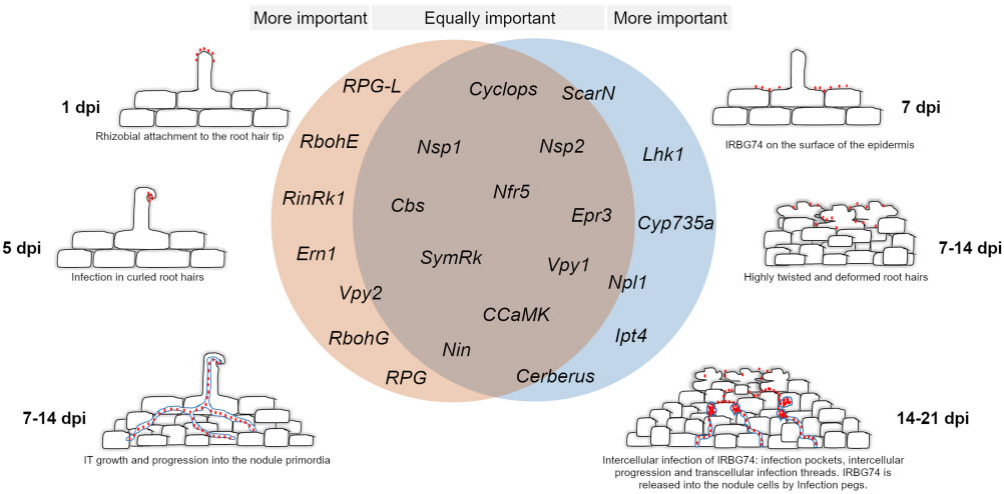
Comparative model of gene requirements in the intracellular and intercellular symbiotic program in *L. japonicus*. A core of essential genes is recruited by *L. japonicus* for root nodule symbiosis, regardless the type of infection mechanism employed by rhizobia. Nonetheless, certain players are particularly more relevant depending on the mode of colonization. Intercellular infection in *L. japonicus* appears to be more sensitive to the absence of certain cytokinin-related genes. IRBG74 colonizes *L. japonicus* roots intercellularly, provoking massive root hair deformation. The intercellular progression of IRBG74 is followed by transcellular and infection peg in the nodule cells. Red dots: *M. loti* (left panel) and IRBG74 (right panel).

In the *Lotus*–*M. loti* symbiosis, the cytokinin receptor mutant *lhk1* shows a delayed and reduced nodulation (Murray et al., 2007), whereas the *ipt4* and *cyp735a* mutants show minor or insignificant phenotypes, respectively (Reid et al., 2017; Supplemental Fig. 6). The RNAseq data presented in this study indicates that these genes are differentially regulated by *M. loti* or IRBG74. In order to determine the relevance of these cytokinin-related genes in the *Lotus*-IRBG74 symbiosis, nodulation kinetics at 1–6 wpi were scored for the *cyp735a, ipt4*, and *lhk1* mutants after IRBG74 inoculation. The nodulation capacity of both *cyp735a* and *ipt4* mutants was substantially reduced, although with different symbiotic phenotypes. The *cyp735a* mutant developed similar numbers of nodules to w. t. plants, at 2 and 3 wpi (Figure 7A;Supplemental Figure 1E), but at 4–6 wpi *cyp735a* formed more nodule-like structures, most of them uninfected white nodules (Figure 7C, right panel). In contrast, at 2–4 wpi, the total number of nodules in *ipt4* was lower compared with w.t. plants (Supplemental Figure 1E). The number of pink nodules was reduced at all timepoints tested and these comprised a mixture of pink and pale pink nodules (Figure 7, A and D, right panel). Interestingly the *lhk1* mutant was unable to develop any nodule structure in the presence of IRBG74 (Figure 7A;Supplemental Figure 1E). This drastic symbiotic phenotype contrasts with the *lhk1* plants inoculated with *M. loti*, where several pink nodules were observed at 6 wpi (Supplemental Figure 6). These results further demonstrate the different cytokinin regulation during intercellular infection in *Lotus*, whereby *Cyp735a*, *Ipt4*, and *Lhk1* are important players for this type of rhizobial infection (Figure 8).

## Discussion

### *Lotus*-IRBG74 symbiosis: a working model to study intercellular infection

Intercellular colonization of legumes by rhizobia occurs via a variety of entry modes. Bacteria can penetrate through middle lamellae of root hairs, cracks at emergent lateral roots or between epidermal cells (Subba-Rao et al., 1995; Gonzalez-Sama et al., 2004; Goormachtig et al., 2004; Bonaldi et al., 2011). Diverse *Lotus* spp. exhibit this infection mechanism, which leads to the formation of either ineffective or nitrogen-fixing nodules, depending on the growth conditions and rhizobial partner (Ranga Rao, 1977; James and Sprent, 1999; Liang et al., 2019). Previously, it was described that under certain mutant background conditions and with a low frequency, *Lotus* roots can be intercellularly colonized by *M. loti* (Madsen et al., 2010) by an NF-independent mechanism. In our study, we have formulated a working model in which intercellular infection can be effectively studied in a model legume, and therefore, our approaches were focused on this process. The compatibility and nodulation rate of the *L. japonicus*–IRBG74 symbiosis represents an optimal system to analyze the intercellular infection, taking advantage of a comprehensive set of methodologies, techniques, and resources (Sandal et al., 2002;  Malolepszy et al., 2016; Mun et al., 2016; Kelly et al., 2018 ; Kamal et al., 2020; Shah et al., 2020). These resources have not been developed for the *Lotus burttii*– *Rhizobium leguminosarum* Norway interaction (Liang et al., 2019), whereas the *L. japonicus*–*R. leguminosarum* Norway approach is excluded by the inability of *R. leguminosarum* Norway to induce nodules on *L. japonicus* (Liang et al., 2018). We show that IRBG74, a nitrogen-fixing strain originally isolated from *Sesbania* spp., massively accumulates on the root surface of *Lotus* roots and invades the roots between the epidermal and root hair cells. This intercellular invasion transforms into transcellular ITs or peg infection structures, which allows the gene-dependencies of these processes to be distinguished from the genes required for root hair ITs. Intercellularly infection by IRBG74 at lateral root junctions of *Sesbania* spp. has also been observed under flooded conditions (Cummings et al., 2009) and in *S. rostrata*, where the infection pocket, formed by an intercellular invasion of certain *Azorhizobium* spp., is followed by transcellular ITs (Goormachtig et al., 2004). Although IRBG74 promoted nodule formation in *Lotus*, the organogenesis and infection programs were delayed, compared with the symbiotic proficient *M. loti*. This was evidenced by a reduced plant growth promotion, compared with that obtained by *M. loti*, which indicates that IRBG74 might not be fully compatible with *Lotus*. The delay could be explained by differences in NF composition and/or abundance, produced by *M. loti* R7A and IRBG74. More than 40 different NF structures are synthesized by IRBG74, mostly pentasaccharides, carbamoylated, and fucosylated, with N-methylation at the nonreducing end (Poinsot et al., 2016). These chemical modifications can be found in the NF synthesized by *M. loti* (Lopez-Lara et al. 1995;  Bek et al., 2010); however, the terminal decorations in the reducing end executed by *nodZ* and *nolL* are particularly relevant, since *Lotus* spp. inoculated with R7AΔ*nodZ* and R7A*ΔnolL* mutants, exhibit delayed nodulation development (Rodpothong et al., 2009). Likewise, the timing could be impacted by differences in the cell surface composition of IRBG74 since production and composition of EPS, LPS, and KPS affect the nodulation kinetics of rhizobial strains in *Lotus* (D'antuono et al., 2005; Townsend et al., 2006; Kelly et al., 2013).

### Common gene dependencies of intercellular and intracellular colonization

The characterization of several legume mutants has identified the molecular players of the symbiotic pathway, from the early signaling to nodule organogenesis. NF receptors are indispensable for initiating symbiotic signaling, since *Lotus* mutants disrupted in these genes do not show any symbiotic response (Madsen et al., 2003; Radutoiu et al., 2003). The nodulation tests of *nfr5* with IRBG74 and w.t. plants with an IRBG74-*nodA* mutant revealed that an NF signaling pathway is required for the *Lotus*–IRBG74 symbiosis. Likewise, both intercellular rhizobial infection and nodule organogenesis is an NF-dependent process in the *S. rostrata*–*A. caulinodans* relationship (Capoen et al., 2010), but there are also intercellular processes whereby an NF-independent mechanism can lead to nitrogen-fixing nodules in certain legumes (Giraud et al., 2007; Madsen et al., 2010; Ibañez and Fabra, 2011). This study revealed a genetic machinery that is equally important for both types of infection modes, since a similar detrimental impact on the nodulation process was observed in the *nfr5, symrk, ccamk, cyclops, nin, nsp1, nsp2 epr3, cbs*, and *vpy1* mutants whether *M. loti* or IRBG74 were used as inoculum in *Lotus* (Figure 8). This set of results shows that these genes participate in the formation of root hair ITs, transcellular ITs, and peg structures. However, the intracellular symbiotic program was more affected in the *rinrk1, ern1, rbohE, rbohG, rpg, rpg-like*, and *vpy2* mutants. One interpretation is that these genes are more important for formation of root hair ITs than transcellular ITs and peg structures. Previously, it was described in *A. evenia* that SYMRK, CCaMK, and the HK1 were required both in the intracellular and intercellular infection by *Bradyrhizobium* (Fabre et al., 2015), reinforcing the notion of a common genetic repertoire for these types of rhizobial infection. Likewise, SYMRK and CCaMK are required for intercellular infection and nodulation in the IRBG74–*Lotus* symbiosis. However, intercellular colonization is apparently more sensitive to the absence of certain genes. *cerberus*, *npl1*, and *scarN* mutants developed numerous white nodules, frequently uninfected after *M. loti* inoculation (Yano et al., 2009; Xie et al., 2012; Qiu et al., 2015), but when IRBG74 was used as inoculum, nodule development and the number of pink nodules were severely reduced in these mutants. This suggests that actin rearrangement plays an important role in formation of cortical and transcellular ITs and that initiation of ITs from intercellular infection pockets is more dependent on actin rearrangement.

As mentioned above, there are few reports describing the molecular components required for intercellular infection. One of them, revealed the positive role of ROS to induce cell death during the crack entry infection of *Azorhizobium* in *S*. *rostrata*. Deprivation of ROS production by applying diphenyleneiodonium chloride, an inhibitor of the ROS-producing enzymes RBOHs, prevents rhizobial colonization in this legume (D'Haeze et al., 2003). Similarly, these genes have been implicated in the IT development during intracellular rhizobial infection in *Medicago* and *P. vulgaris* (Peleg-Grossman et al., 2007; Montiel et al., 2012; Arthikala et al., 2017). Likewise, the nodulation program was delayed in *Lotus* mutants disrupted in *RbohE* or *RbohG* genes after inoculation with *M. loti*. The intercellular symbiotic performance by IRBG74 was not negatively impacted in the *rbohE* and *rbohG* mutants, which indicates that ROS produced by these isoforms is not relevant in this process, however since the *LjRboh* gene family is composed by nine members (Montiel et al., 2018), other isoforms could contribute to the intercellular infection by IRBG74. Inhibition of ethylene synthesis or perception has a negative effect on the nodulation and intercellular infection induced by *Azorhizobium* in *S. rostrata* (D'Haeze et al., 2003). In contrast, ethylene plays a negative role in the *Lotus*–*M. loti* symbiosis. The *ein2a ein2b* double mutant that exhibits complete ethylene insensitivity is hyperinfected and hypernodulated by *M. loti* (Reid et al., 2018). Unlike, the intercellular symbiotic process in *S. rostrata*, where ethylene is required for nodulation, the *ein2a ein2b* mutant was hypernodulated by IRBG74, indicating that this phytohormone is not essential for the IRBG74 intercellular infection. Consequently, the positive role of ethylene in the intercellular infection is not conserved in *Lotus*, which highlights the relevance of a better understanding of the intercellular colonization in legumes.

### Distinctive cytokinin signaling program during intercellular infection

The dual role of cytokinins, as positive and negative regulators of nodule development and rhizobial infection, respectively, makes them key phytohormones in the legume–rhizobia symbiosis (Miri et al., 2016). The *lhk1* mutant belatedly develops a reduced number of pink nodules in response to *M. loti* infection, but when IRBG74 is used as inoculum the mutant is unable to form nodules up to 6 wpi. In the intracellular infection mediated by *M. loti*, it has been suggested that other cytokinin receptors are sufficient to induce nodule organogenesis in the absence of *Lhk1* (Murray et al., 2007). However, the signaling pathway triggered by LHK1 is indispensable in the *Lotus*–IRBG74 symbiosis. Conversely, the *ern1* mutant displayed improved symbiotic performance with IRBG74, which further confirms that depending on the type of rhizobial infection program, distinct signaling pathways are triggered. This was reflected in the different transcriptomic responses of genes involved in the synthesis, perception signaling and metabolism of cytokinin induced by *M. loti* or IRBG74. It has been shown that different cytokinin biosynthesis genes are induced during intracellular colonization of *M. loti* in *Lotus* roots (Reid et al., 2016, 2017). Although the cytokinin *trans*-hydroxylase *Cyp735a* is highly induced by *M. loti*, the nodulation performance is not significantly affected in *Lotus* plants disrupted in this gene (Reid et al., 2017). In contrast, nodule organogenesis is delayed and reduced in *cyp735a* mutants inoculated with IRBG74. *Cyp735a* encodes cytochrome P450 monooxygenases (P450s) that catalyze the biosynthesis of trans-Zeatin, an isoprenoid cytokinin compound (Takei et al., 2004), indicating that tZ cytokinins play a more relevant role during intercellular colonization. The mutant affected in the isopentenyl transferase 4 (*Ipt4*) gene showed a mild impact on the nodulation capacity with *M. loti* (Reid et al., 2017). However, in response to IRBG74 inoculation, the development of nodules was evidently delayed, and the number of pink nodules reduced. IPT is placed in the first step during isoprenoid cytokinin biosynthesis, giving rise to iP riboside 50-diphosphate or iP riboside 50-triphosphate intermediates, which can be converted by CYP735a to tZ cytokinins (Hwang and Sakakibara, 2006). The delayed nodulation and enhanced phenotypes of the cytokinin-related mutants may indicate that the peak of cytokinin triggered by the intercellular program is lower or more dispersed in the root relative to the highly localized cytokinin signaling achieved in the intracellular infection modes.

## Conclusions

The intracellular colonization has been extensively described at the cellular and molecular levels in recent decades, but there is little knowledge about the molecular players controlling the intercellular invasion. In this study, we find that *Lotus* has a genetic repertoire that confers on it the ability to establish effective symbiotic processes with rhizobia, both intracellularly and intercellularly, using a core genetic machinery. However, there is a set of molecular players that are dispensable or recruited, depending on the mode of infection. Different approaches revealed that the cytokinin signaling pathway is apparently a key difference to be further analyzed. Similarly, other components seem to be differentially relevant for this type of infection. For instance, the *rinrk1* receptor mutant showed a better nodulation performance with IRBG74 compared with *M. loti*. The latter indicates that during intercellular infection, certain uncharacterized ligands are not entirely necessary for rhizobial colonization. This study opens new research questions, for instance, the identification of novel players exclusively required for intercellular infection using the *Lotus* mutant collections. A comparative analysis of the molecular components required for the different intercellular colonization modes in Robinoid, Genistoid, and Dalbergoid legumes would also be interesting and might contribute to our understanding of symbiosis from an evolutionary perspective. Although the plant genes are crucial for this process, the identification of the bacterial genes promoting intercellular infection would have an impact on microbiome studies.

## Materials and methods

### Germination and nodulation assays

*Lotus* seeds of accession Gifu (Handberg and Stougaard, 1992) were scarified with sandpaper, surface disinfected with 0.3% (v/v) of sodium hypochlorite for 10 min and then washed five times with autoclaved distilled water, to remove traces of chlorine. The washed seeds were incubated overnight at 4°C and then transferred to square Petri dishes for germination at 21°C. For monitoring nodulation kinetics, 3-d postgermination seedlings (*n* ≥ 20 plants per condition) were placed into square Petri dishes with 1.4% B&D agar slant covered with filter paper and inoculated with the respective rhizobial strains (1 mL of bacterial culture per plate; OD_600_ = 0.05). Through this method, *Lotus* roots were kept in contact with the inoculum throughout the experiment. After rhizobial inoculation the number of white (bumps and nodule primordia) and pink nodules were recorded weekly until 6 wpi with a stereomicroscope. The plants were harvested at 6 wpi to measure the fresh weight and length of the aerial part. LORE1 lines (Urbanski et al., 2012; Malolepszy et al., 2016) disrupted in the genes of interest were ordered from the LORE1 database (https://lotus.au.dk/) and genotyped with allele-specific primers (Supplemental Table 5) to obtain homozygous mutants following the database instructions (Mun et al., 2016). The gene IDs and the corresponding LORE1 IDs are shown in Supplemental Table 1. Both the w.t. and mutant plants used in this study are in the Gifu accession, except for the lines MG-134, MG-144, and MG-145 used in Supplemental Figure 1.

### Infection phenotyping using confocal microscopy

Three-day-old seedlings of *Lotus* (accession Gifu; Handberg and Stougaard, 1992) were placed on 1/4 B&D plates and inoculated with fluorescently labelled *M. loti* R7A (Kelly et al., 2013) or IRBG74 strains (Cummings et al., 2009), obtained through transformation with the constitutive DsRED expressing plasmid pSKDSRED (Kelly et al., 2013). The roots were harvested from the plates at different time points. To enable observations in deeper parts of the tissue, a fluorescent compatible clearing protocol was used as described before (Nadzieja et al., 2019). Cleared roots were visualized by confocal microscopy with the following excitation lasers/emission cut-offs: 405/408–498 nm (autofluorescence: intensity, 3%–11%; Gain, 570–620), 561/517–635 nm (DsRed: intensity, 5%–12%; Gain, 620–640). For 3D projections, Fiji ImageJ (Schindelin et al., 2012) was used to create animation frames, which then were rearranged using Adobe Photoshop CC into final projections.

### Nodule histology analysis

Young and mature nodules at 3 wpi with IRBG74 or *M. loti* were detached from *Lotus* roots, sliced in half, and incubated overnight in fixative solution (2.5% glutaraldehyde, 0.1 M sodium cacodylate pH 7). The fixed nodules slices were embedded in acrylic resin and sectioned for light and TEM. The nodule slices were stained with Toluidine blue for light microscopy analysis (James and Sprent, 1999; Madsen et al., 2010). The number of transcellular IT, bacteroids per symbiosome and area of the bacteroids were recorded from images taken of 10 slides from 7 different young and mature nodules colonized by IRBG74 or *M. loti*.

### Rhizobial occupancy by qPCR

Four wpi roots (including nodules) were surface disinfected with 0.3% of sodium hypochlorite and 70% EtOH solution for 1 min to remove rhizobia attached to the root surface, and then washed five times with distilled water. Later, DNA was isolated from individual roots (Chomczynski and Rymaszewski 2006) and the concentration adjusted to be used as a template (10 ng/*µ*L). *NodA* abundance from *M. loti* R7A (forward: TATGAGCCGACCGGAGCCTTCAAT and reverse: CCGTATAGACCGAGTTCAGCGACCA) and IRBG74 (forward: GAACTGCAAGTTGACGATCACGC and reverse: AAACGTCGTAACAAGCCCATGTGG) was measured by qPCR. The expression values were normalized to the abundance of the *Lotus* gene (LotjaGi1g1v0152000.1; forward: GAAGGACCCAGAGGATCACA and reverse: CGGTCTTCGTACTTCTTCGC) using the delta Ct method (Pfaffl, 2001).

### RNAseq of *Lotus* roots and bioinformatics

The susceptible infection zone of *L. japonicus* roots by IRBG74 (elongation and maturation zone of the root) was cut and then freezed in liquid nitrogen from seedlings at 3, 5, and 10 dpi with IRBG74 (OD_600_ = 0.05) or mock-treated (water) at the same time points. Total RNA was isolated and DNA contamination was removed by DNAse treatment. Library preparations using randomly fragmented mRNA were performed by IMGM laboratories (Martinsried, Germany) and sequenced in paired-end 150-bp mode on a Illumina NovaSeq 6000 instrument.

A decoy-aware index was built for Gifu transcripts using default Salmon parameters and reads were quantified using the validateMappings flag (Salmon version 0.14.1; (Patro et al., 2017). Normalized expression levels and differential expression testing were performed using the R-package DESeq2 version 1.20 (Love et al., 2014) after summarizing gene level abundance using the R-package tximport (version 1.8.0).

### Statistical analysis

Significant differences of pink and total number of nodules among different inoculums and genetic backgrounds were calculated by Mann–Whitney *U*-test. Other parameters evaluated in this study were determined by Student’s *t* test. The calculated expression values and statistics of the RNAseq data are included as Supplemental Table 4.

### Accession numbers

The RNAseq reads associated with this study are available in the SRA under bioproject accession number PRJNA632725. The *L. japonicus* (accession Gifu and MG20) gene identifiers are shown in Supplemental Table S1.

## Supplemental Data

The following materials are available in the online version of this article.

**Supplemental Figure S1.** Nodulation kinetics in different *L. japonicus* accessions and mutants.

**Supplemental Figure S2.** Nodule cell occupancy and infection in *L. japonicus* nodules colonized by *Mesorhizobium loti* or IRBG74.

**Supplemental Figure S3.** Nodulation performance of *L. japonicus* mutants.

**Supplemental Figure S4.** Ethylene production in *L. japonicus* roots inoculated with rhizobia.

**Supplemental Figure S5.** Differentially expressed genes (DEG) in *L. japonicus* roots after rhizobial inoculation.

**Supplemental Figure S6.** Phenotype of *L. japonicus* mutants at 6 wpi with *Mesorhizobium loti* or IRBG74.

**Supplemental Table S1.** List of *L. japonicus* mutants used in this study.

**Supplemental Table S2.** Mutants with a Nod-phenotype in response to *Mesorhizobium loti* or IRBG74 inoculation.

**Supplemental Table S3.** Number of ITs per plant at 1 wpi with *Mesorhizobium loti*.

**Supplemental Table S4.** RNAseq expression data of *L. japonicus* roots inoculated with *Mesorhizobum loti* (1 and 3 dpi) or IRBG74 (3, 5, and 10 dpi).

**Supplemental Table S5.** List of primers used for genotyping.

**Supplemental Movie S1.** 3D-projection of *L. japonicus* roots infected by IRBG74 at 1–3 wpi.

## Supplementary Material

kiaa049_Supplementary_Data

## Notes

J.M., D.R., C.M.B., and T.H.G. characterized and isolated the mutants. D.R. and J.M. processed and analyzed the RNAseq data, respectively. M.N., F.A.D., and E.K.J. performed the microscopy analyses. J.M. wrote the manuscript and prepared the figures with contributions of the co-authors. J.M., S.K., T.O., and J.S. conceived the research plan. J.S. and S. K. coordinated and guided the research.

The author responsible for distribution of materials integral to the findings presented in this article in accordance with the policy described in the Instructions for Authors (https://academic.oup.com/plphys) is: Jens Stougaard (stougaard@mbg.au.dk).
