# Supplementary material for: Distinct signaling routes mediate intercellular and intracellular rhizobial infection in Lotus japonicus
Source: Plant Physiol. 2020 Dec 4;185(3):1131–47. doi: 10.1093/plphys/kiaa049 (PMC8133683; doi:10.1093/plphys/kiaa049)
Supplement: kiaa049_Supplementary_Data [file kiaa049_supplementary_data.zip › pp.01566.2020-s06.pdf]

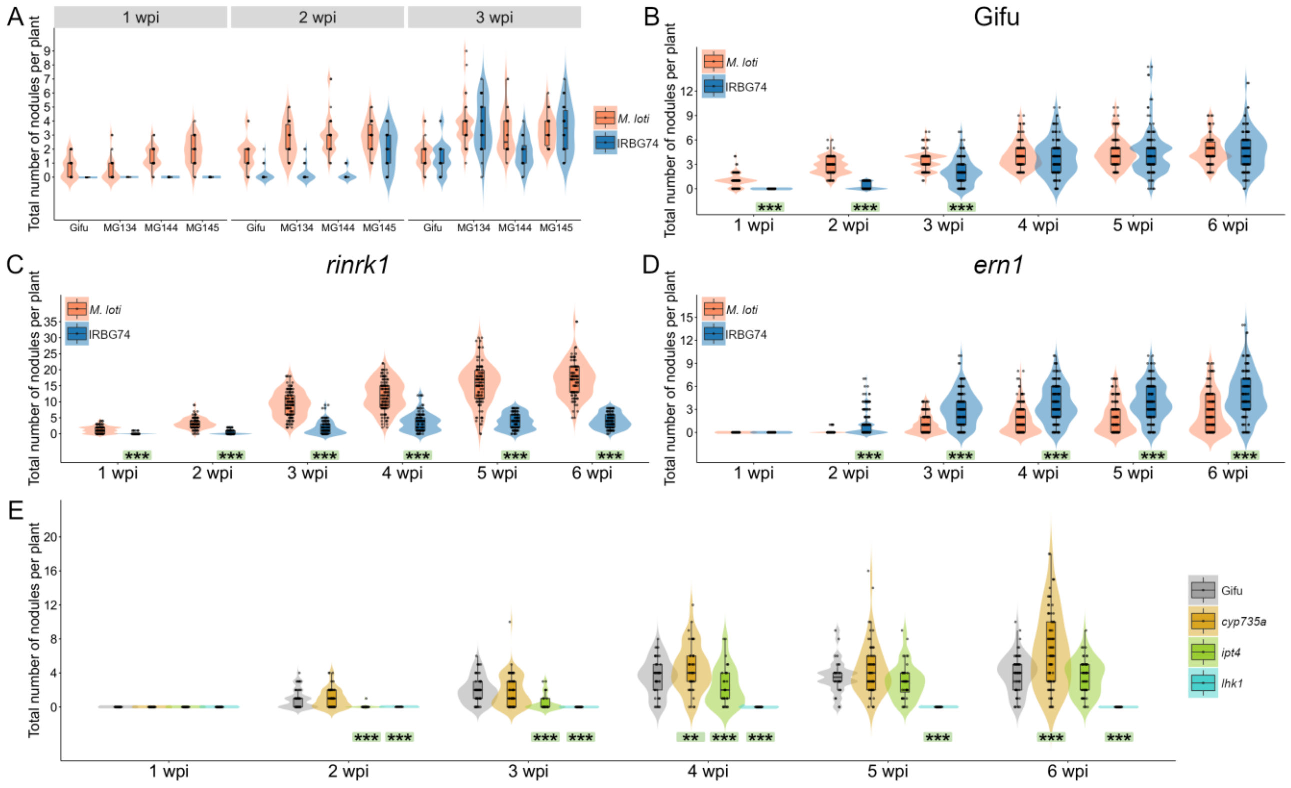

Supplemental figure S1. Nodulation kinetics in different *Lotus japonicus* accessions and mutants. A) Nodulation kinetics of Gifu, MG134, MG144 and MG145 *Lotus* accessions at 1-3 wpi with *M. loti* and IRBG74. Total number of nodules at 1-6 wpi with *M. loti* (B-D) or IRBG74 (B-E) in wild type Gifu (B and E), *rinrk1* (C), *ern1* (D), *cyp735a*, *ipt4* and *lhk1* (E) mutants. Mann Whitney U test of total number of nodules (asterisks below the violin graphs indicates significant differences) between *Lotus* plants inoculated with *M. loti* and IRBG74 (A-D) or between Gifu and mutants colonised by *cyp735a*, *ipt4* and *lhk1* (E). P-values < 0.05 and 0.001 are marked with one or two asterisks, respectively. Violin boxplots: center line, median; box limits, upper and lower quartiles; whiskers, 1.5x interquartile range; points, individual data points.

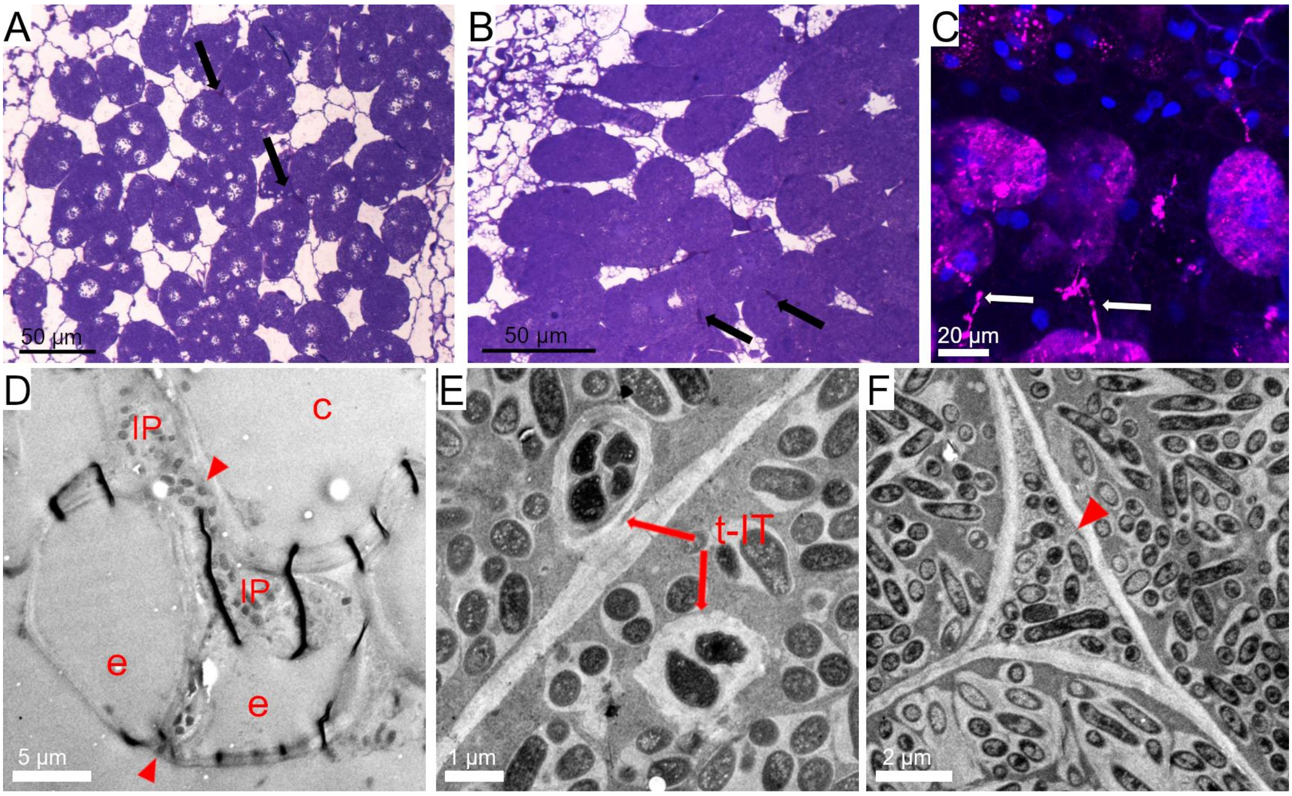

Supplemental Figure S2. Nodule cell occupancy and infection in *Lotus japonicus* nodules colonized by *Mesorhizobium loti* or IRBG74. Infected cells in mature (A, B, C and F) and young (D and E) nodules of *Lotus* at 3 wpi with *M. loti* (A) or IRBG74 (B-F). C, Section of a *Lotus* nodule at 20 dpi with IRBG74-DsRed (magenta) visualized by confocal microscopy. D, Intercellular infection between the epidermal cells and infection pockets (IP) in a young nodule colonized by IRBG74. Progression of IRBG74 occurs by transcellular infection threads (t-IT) and intercellular infection, indicated with arrows and arrowheads, respectively. Black lines in D are artifacts caused by folding during the sectioning.

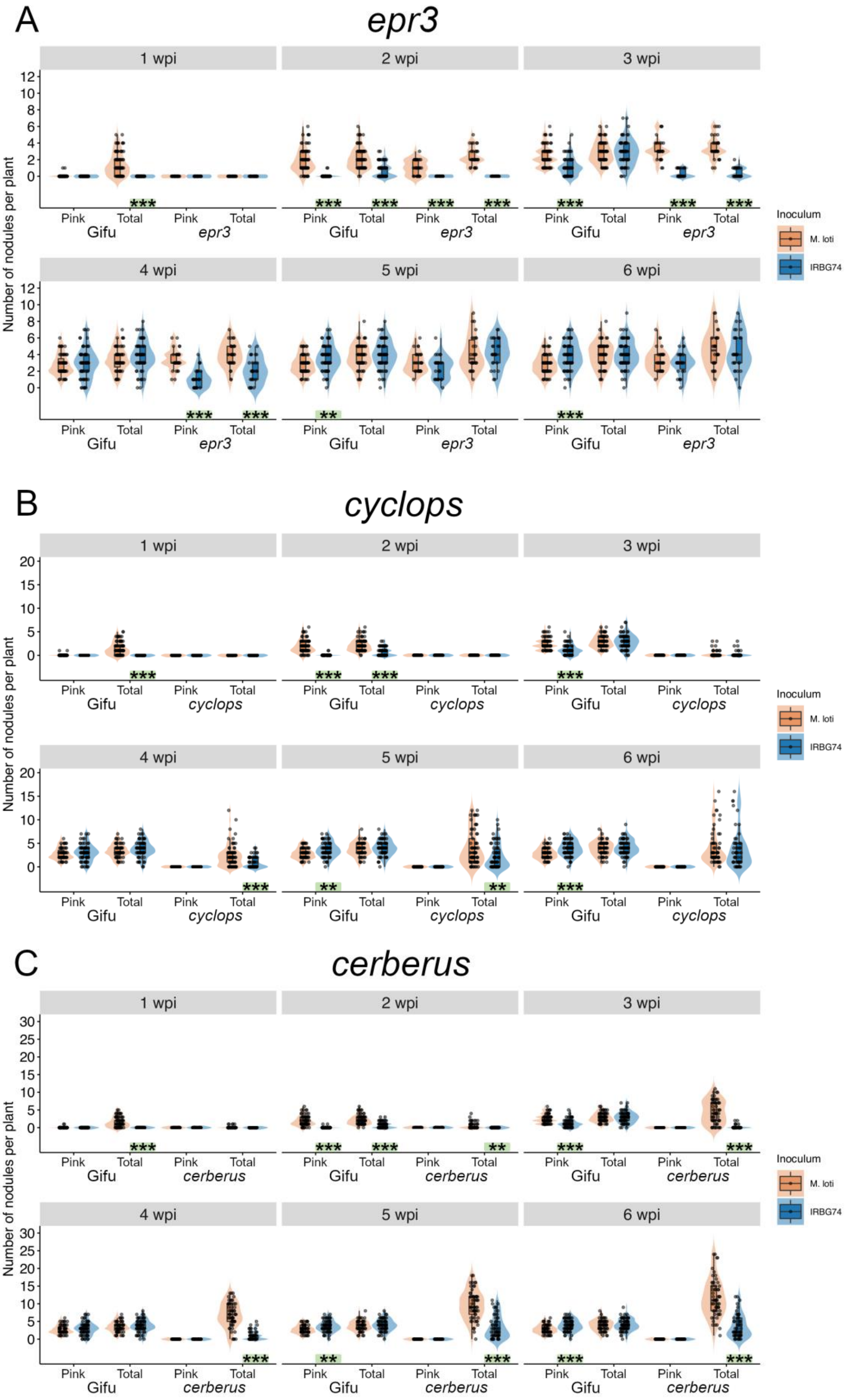
A
epr3
1 wpi
2 wpi
3 wpi
4 wpi
5 wpi
6 wpi
Number of nodules per plant
Pink
Total
Gifu
epr3
Inoculum
M. loti
IRBG74
B
cyclops
1 wpi
2 wpi
3 wpi
4 wpi
5 wpi
6 wpi
Number of nodules per plant
Pink
Total
Gifu
cyclops
Inoculum
M. loti
IRBG74
C
cerberus
1 wpi
2 wpi
3 wpi
4 wpi
5 wpi
6 wpi
Number of nodules per plant
Pink
Total
Gifu
cerberus
Inoculum
M. loti
IRBG74

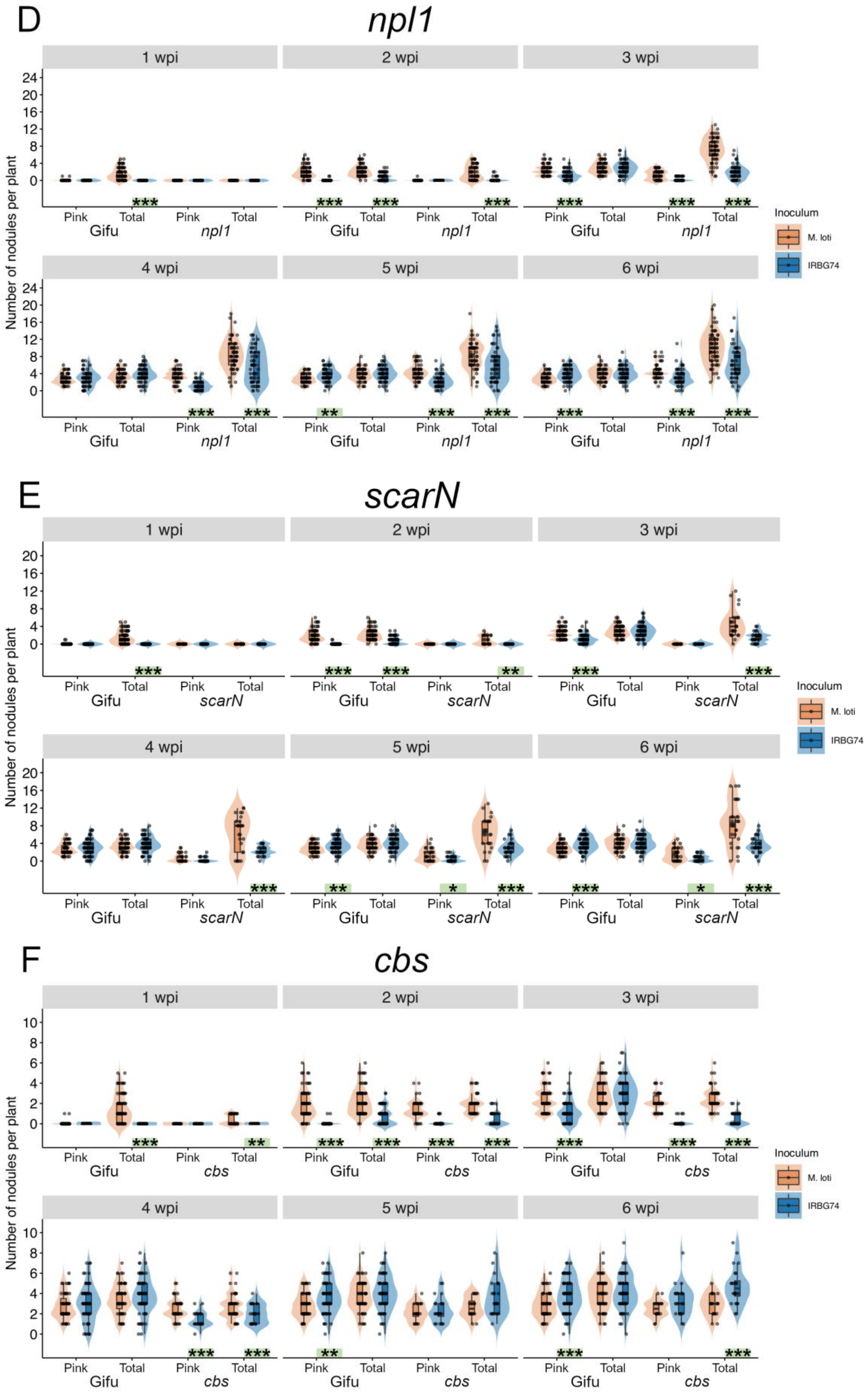

D
npl1
1 wpi
2 wpi
3 wpi
4 wpi
5 wpi
6 wpi
Number of nodules per plant
Pink
Total
Gifu
npl1
Inoculum
M. loti
IRBG74
E
scarN
1 wpi
2 wpi
3 wpi
4 wpi
5 wpi
6 wpi
Number of nodules per plant
Pink
Total
Gifu
scarN
Inoculum
M. loti
IRBG74
F
cbs
1 wpi
2 wpi
3 wpi
4 wpi
5 wpi
6 wpi
Number of nodules per plant
Pink
Total
Gifu
cbs
Inoculum
M. loti
IRBG74

G *vpy1*

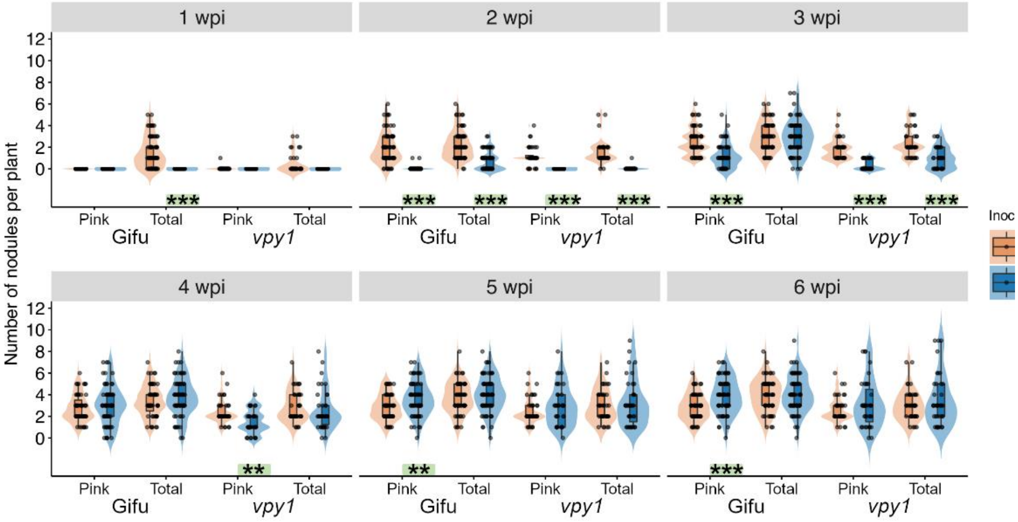

I *rpg*

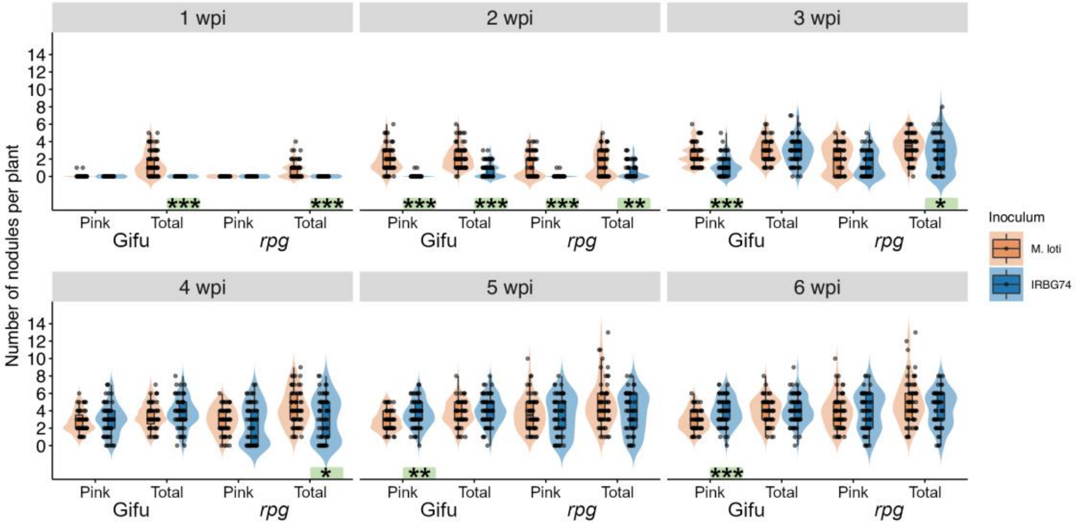

J *rpg-like*

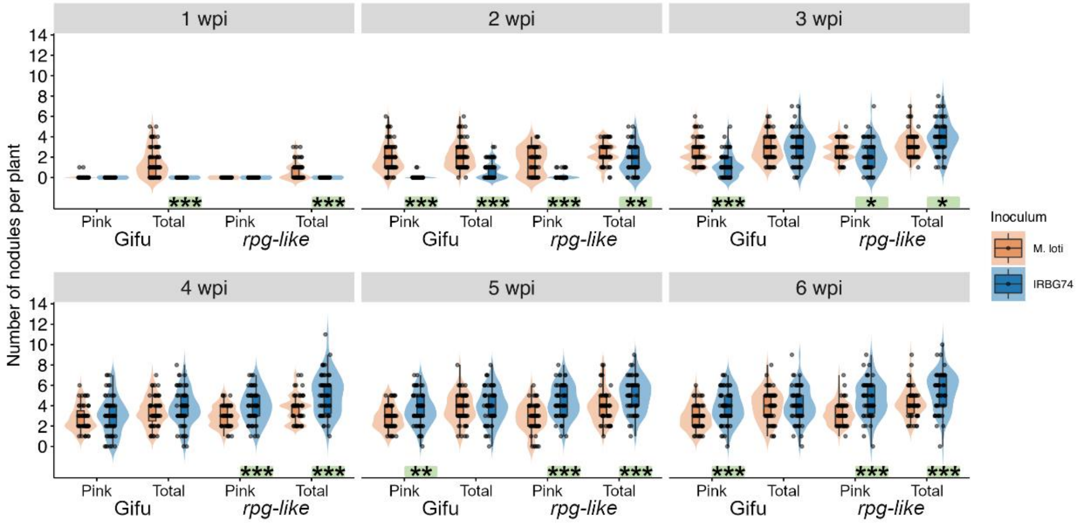

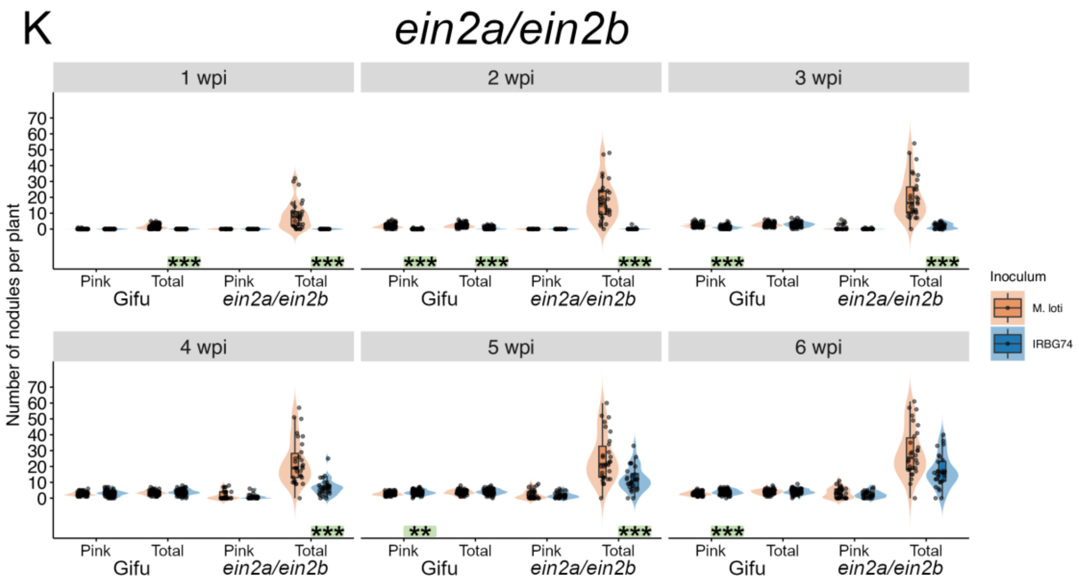

Supplemental Figure S3. Nodulation performance of *Lotus japonicus* mutants. Nodulation kinetics of *Lotus* plants from 1 to 6 wpi with *M. loti* or IRBG74. Error bars mean SE. Student's *t*-test analyses of pink and total number of nodules (red and black asterisks, respectively) between plants inoculated with *M. loti* or IRBG74 in the same genetic background. P-values < 0.05, 0.01 and 0.001 are marked with one, two or three asterisks, respectively. Violin boxplots: center line, median; box limits, upper and lower quartiles; whiskers, 1.5x interquartile range; points, individual data points.

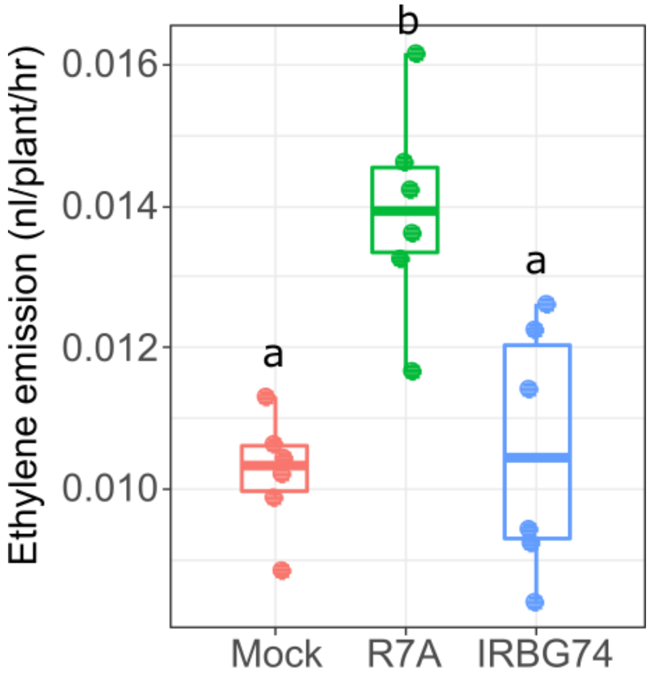

Supplemental Figure S4. Ethylene production in *Lotus japonicus* roots inoculated with rhizobia. Ethylene emission of *Lotus* roots over first 24 h after mock treatment (mock: red) or inoculation with *M. loti* (R7A: green) and IRBG74 (blue). Measurements performed as described by Reid et al. (2018). Letters represent statistical differences determined by Tukey test at $P < 0.05$. Boxplots: center line, median; box limits, upper and lower quartiles; whiskers, 1.5x interquartile range; points, individual data points.

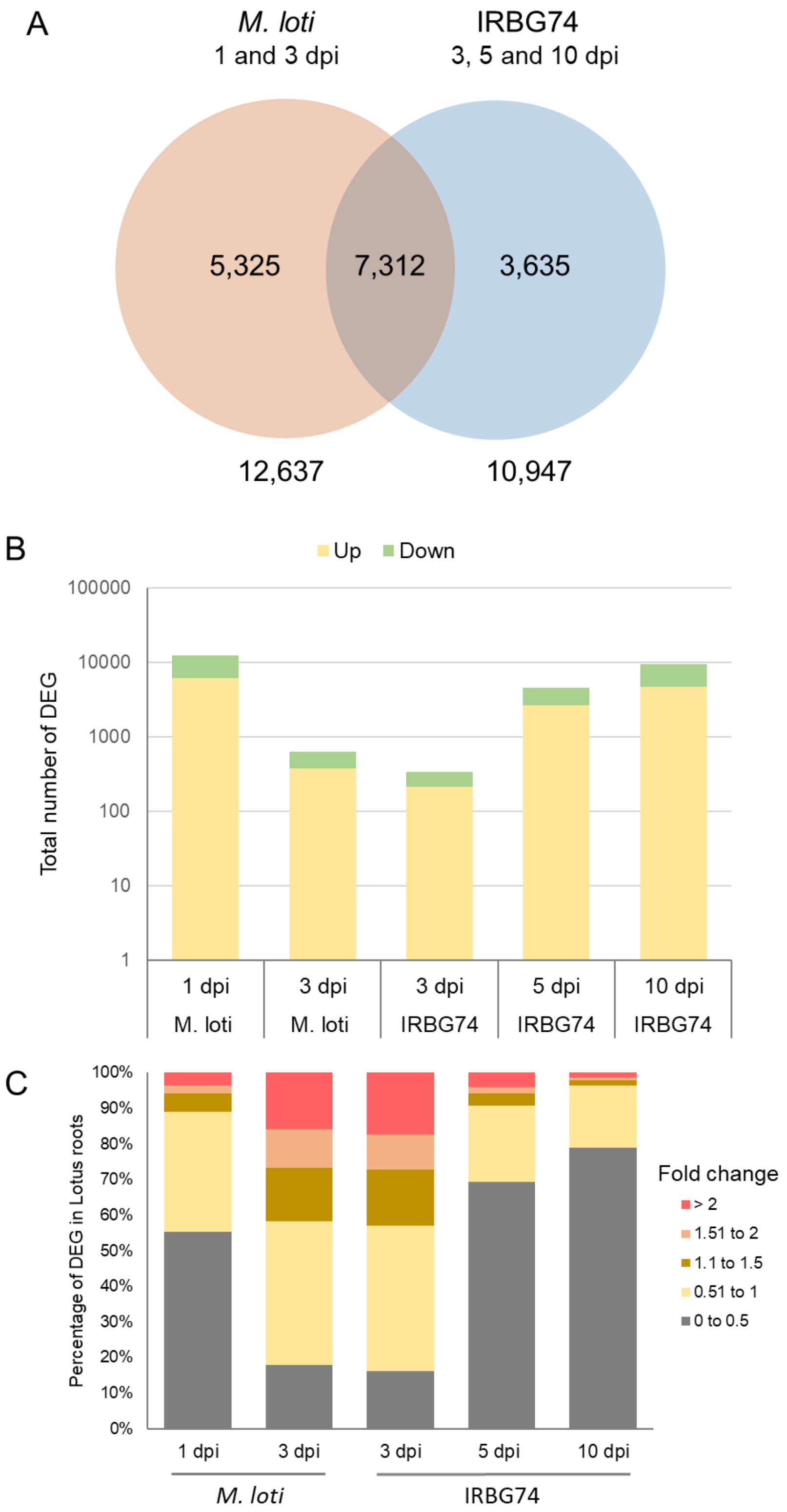

Supplemental Figure S5. Differentially expressed genes (DEG) in *Lotus japonicus* roots after rhizobial inoculation. A, Venn diagram with total DEG by *M. loti* (1 and 3 dpi) and IRBG74 (3, 5 and 10 dpi) colonization. B, Total number of DEG (Up and down-regulated) at different time points after *M. loti* or IRBG74 inoculation. C, Proportion of DEG with different fold change levels at different time points with *M. loti* or IRBG74. P-adjust < 0.5

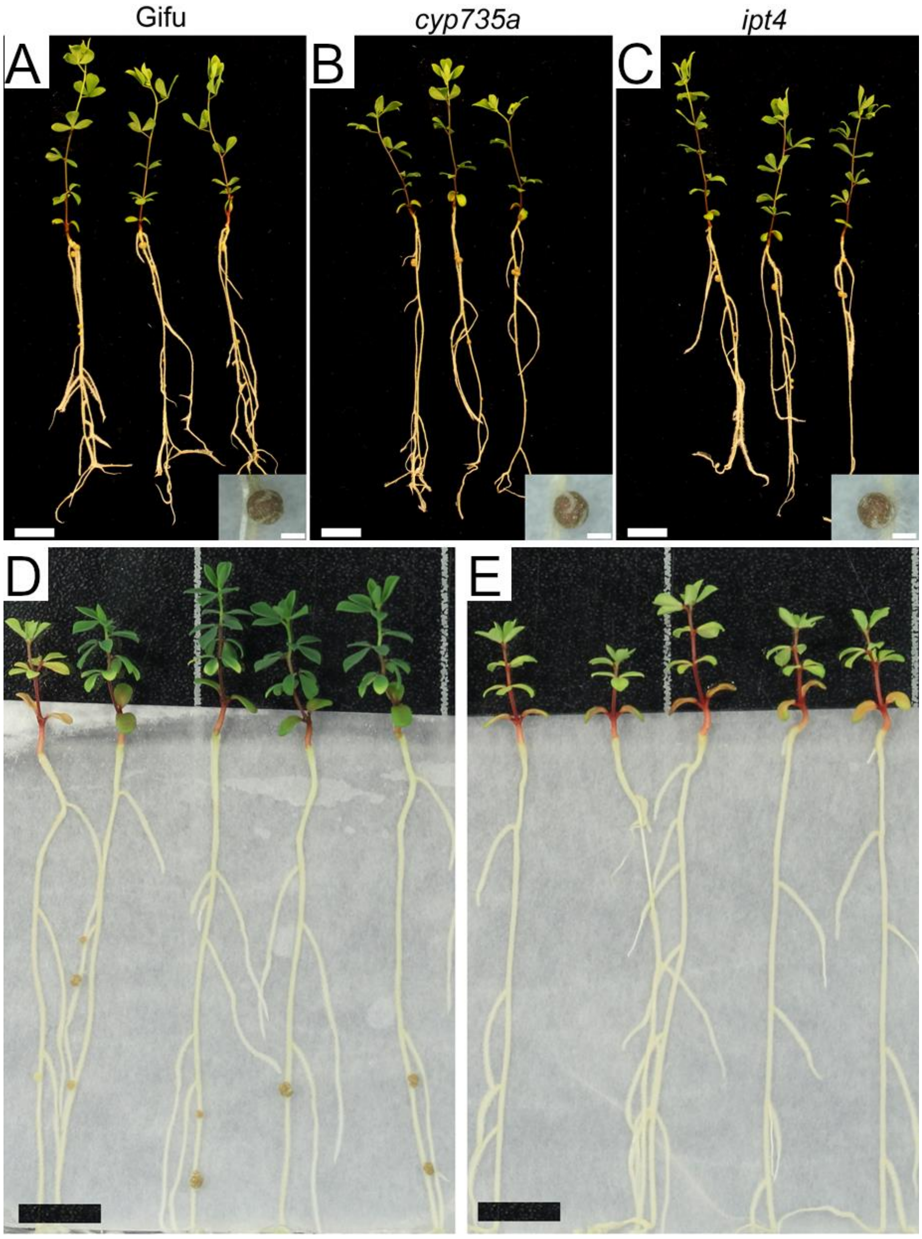

Supplemental Figure S6. Phenotype of Lotus japonicus mutants at 6 weeks post-inoculation (wpi) with *Mesorhizobium loti* or IRBG74. Representative images of Gifu (A), *cyp735a* (B), *ipt4* (C) and *lhk1* (E and F) mutants inoculated with *M. loti* (D) or IRBG74 (A-C and E). Scale bar, 1 cm. Insets in A-C show nodules at 5 wpi in the respective genetic backgrounds. Scale bar, 1 mm.

Supplemental Table S1. List of *Lotus japonicus* mutants used in this study.

Mutant	Gene ID MG20	Gene ID GIFU	LORE1 ID	Reference
cbs	Lj6g3v1537040	LotjaGi2g1v0126500	30034737	This work
cerberus	Lj5g3v1697430	LotjaGi5g1v0256800		Yano et al. 2009
cyclops-2	Lj2g3v1549600	LotjaGi2g1v0343300		Yano et al. 2008
cyp735a	Lj1g3v3317470	LotjaGi1g1v0550400		Reid et al. 2017
ein2a	Lj1g3v4590690	LotjaGi1g1v0690600		Reid et al. 2018
ein2b	Lj5g3v0659810	LotjaGi5g1v0079800		Reid et al. 2018
epr3	Lj2g3v1415410	LotjaGi2g1v0324000		Kawaharada et al. 2015
ern1	Lj1g3v3975310	LotjaGi1g1v0643700		Cerri et al. 2017
ipt4	Lj0g3v0154359	LotjaGi6g1v0298900		Reid et al. 2017
lhk1-1	Lj4g3v3113830	LotjaGi4g1v0459800		Murray et al. 2007
nfr5	Lj2g3v1828350	LotjaGi2g1v0394950		Madsen et al. 2003
nin-2	Lj2g3v3373110	LotjaGi1g1v0001500		Schauser et al. 1999
npl-1	Lj4g3v1389340	LotjaGi4g1v0225600		Xie et al. 2012
nsp1	Lj3g3v2579340	LotjaGi3g1v0414350		Heckmann et al. 2006
nsp2	Lj1g3v0785930	LotjaGi1g1v0257100		Heckmann et al. 2006
rbohE-1	Lj5g3v1497840	LotjaGi5g1v0224200	30014558	This work
rbohG-1	Lj6g3v1549190	LotjaGi5g1v0771200	30013609	This work
rinrk1	Lj4g3v1535150	LotjaGi4g1v0238900		Li et al. 2019
rpg	Lj5g3v1699100	LotjaGi5g1v0253300	30090568	This work
rpg-L-4	Lj5g3v0681160	LotjaGi5g1v0086600	30163956	This work
scar-N	Lj3g3v1393790	LotjaGi3g1v0202300		Qiu et al. 2015
symrk-3	Lj2g3v1467920	LotjaGi2g1v0330500		Madsen et al. 2010
vpy1-1	Lj0g3v0049599	LotjaGi2g1v0091200	30134930	This work
vpy2-1	Lj1g3v3975850	LotjaGi1g1v0646300	30157254	This work

Supplemental Table S2. Mutants with a Nod- phenotype in response to *Mesorhizobium loti* or IRBG74 inoculation.

**Mutant**	**Strain**	**1 wpi**		**2 wpi**		**3 wpi**		**4 wpi**		**5 wpi**		**6 wpi**		**n =**
		**P**	**W**	**P**	**W**	**P**	**W**	**P**	**W**	**P**	**W**	**P**	**W**	
nfr5	*M. loti*	0	0	0	0	0	0	0	0	0	0	0	0	60
	IRBG74	0	0	0	0	0	0	0	0	0	0	0	0	60
symrk	*M. loti*	0	0	0	0	0	0	0	0	0	0	0	0	30
	IRBG74	0	0	0	0	0	0	0	0	0	0	0	0	30
ccamk	*M. loti*	0	0	0	0	0	0	0	0	0	0	0	0	50
	IRBG74	0	0	0	0	0	0	0	0	0	0	0	0	50
nin-2	*M. loti*	0	0	0	0	0	0	0	0	0	0	0	0	45
	IRBG74	0	0	0	0	0	0	0	0	0	0	0	0	69
nsp1	*M. loti*	0	0	0	0	0	0.02	0.02	0.02	0.04	0.04	0.04	0.04	50
	IRBG74	0	0	0	0	0	0	0	0	0	0	0	0	60
nsp2	*M. loti*	0	0	0	0	0	0	0	0	0	0	0	0	18
	IRBG74	0	0	0	0	0	0	0	0	0	0	0	0	16
Gifu (w. t.)	IRBG74-*nodA*	0	0	0	0	0	0	0	0	0	0	0	0	20

P= Pink Nodules

W= White Nodules

Supplemental Table S3. Number of infection threads (ITs) per plant at 1 week post-inoculation (wpi) with *Mesorhizobium loti*.

Genetic background	IT per plant	SE	n=
Gifu	72.6	4.9	15
vpy1	37.2	3.1	16
vpy2	37.2	2.2	17
rpg	33.5	3.8	15

Supplemental Table S5. List of primers used for genotyping.

Mutant	LORE1 ID	Fw Primer	Rv Primer
cbs	30034737	CGTGGTTGTCCATCATGCTTTTGGC	GCAGCCATGTGGCTTTGTCCCTTC
rbohE-1	30014558	TCCGTGAATGACAAGAAGCACATAGACA	CATTTGATGACAACGTCAACTTTCACAAGG
rbohG-1	30013609	TCATGGTCAGAGACATCGAGCACTGG	AGCGCGTTTTGGGGAGTTTTCCCT
rpg	30090568	GACGCTCAAGCTCCAAGTTCCCGA	TGATTGATGTGGAGGGACTGCGGA
rpg-L-4	30163956	TCACCAACAATTTCTCACTCAAATTTGTCC	TGACAGAGAAATCCCGAAAGATCAACAGGA
vpy1-1	30134930	TGGCGCTGTCGATGAAGACTTGCT	CCCTCGCGCGTGTTCCCTATCTCT
vpy2-1	30157254	TGGAGTTGGCGTGTTCATTGGGTG	AATGCAGCGCCGTGTAACCGTCTC
