# Supplementary figures and images for: Distinct signaling routes mediate intercellular and intracellular rhizobial infection in Lotus japonicus
Source: Plant Physiol. 2020 Dec 4;185(3):1131–47. doi: 10.1093/plphys/kiaa049 (PMC8133683; doi:10.1093/plphys/kiaa049)

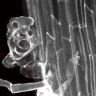

Supplement: kiaa049_Supplementary_Data [file kiaa049_supplementary_data.zip › pp.01566.2020-s07.jpg]
